# Leverage Surface Chemistry for High-Performance Triboelectric Nanogenerators

**DOI:** 10.3389/fchem.2020.577327

**Published:** 2020-11-20

**Authors:** Jing Xu, Yongjiu Zou, Ardo Nashalian, Jun Chen

**Affiliations:** Department of Bioengineering, University of California, Los Angeles, Los Angeles, CA, United States

**Keywords:** surface chemistry, surface engineering, triboelectric nanogenerator, wearable electronics, Internet of Things

## Abstract

Triboelectric Nanogenerators (TENGs) are a highly efficient approach for mechanical-to-electrical energy conversion based on the coupling effects of contact electrification and electrostatic induction. TENGs have been intensively applied as both sustainable power sources and self-powered active sensors with a collection of compelling features, including lightweight, low cost, flexible structures, extensive material selections, and high performances at low operating frequencies. The output performance of TENGs is largely determined by the surface triboelectric charges density. Thus, manipulating the surface chemical properties via appropriate modification methods is one of the most fundamental strategies to improve the output performances of TENGs. This article systematically reviews the recently reported chemical modification methods for building up high-performance TENGs from four aspects: functional groups modification, ion implantation and decoration, dielectric property engineering, and functional sublayers insertion. This review will highlight the contribution of surface chemistry to the field of triboelectric nanogenerators by assessing the problems that are in desperate need of solving and discussing the field's future directions.

## Introduction

The rapid development of wearable and portable electronic devices is greatly revolutionizing our conventional means of energy generation and consumption (Gubbi et al., 2013; Lee and Lee, 2015; Zhou et al., 2020a; Zou et al., 2020). Miniaturized energy sources with high portability and sustainability are eagerly desired for powering billions of distributed devices in the era of the Internet of Things (Bai et al., 2014; Yang et al., 2015; Lin et al., 2017; Xu et al., 2017; Bedi et al., 2018; Wang, 2019). In the modern world, portable energy storage units, such as batteries, seem like the intuitive and most widely used solution to meet the power consumption needs of electronic devices (Grey and Tarascon, 2017; Gu et al., 2017; Liu W. et al., 2017; He et al., 2018; Zan et al., 2020). However, their limited lifetime (Ponrouch et al., 2016; Placke et al., 2017; Liu K. et al., 2018; Wan et al., 2019; Xu et al., 2019), rigid structure, toxic chemical components, and unsustainable working mode, which includes periodically recharging or even replacing the battery unit, deems portable energy storage units obsolete for wide-range adoption to mobile electronics, and more specifically wearable devices (Wang, 2013; Zang et al., 2015; Gao et al., 2016; Kenry and Lim, 2016; Trung and Lee, 2016; Liu Y. et al., 2017; Seneviratne et al., 2017; Gür, 2018; Kim et al., 2019; Yan et al., 2020; Zhang et al., 2020) and bio-integrated applications (Kang et al., 2013; Slater et al., 2013; Li and Dai, 2014; Yabuuchi et al., 2014; Fu et al., 2017; Zhang et al., 2017; Lin et al., 2018; Yan et al., 2018; Meng et al., 2019; Zhou et al., 2020b). Converting the accessible, renewable energy from the human body and its surroundings into electricity is considered a great alternative solution (Wang Z. L. et al., 2015; Chen et al., 2020; Su et al., 2020). Electricity generation from biomechanical motions (Qin et al., 2008; Sun et al., 2011; Lee et al., 2012; Yang W. et al., 2013; Yi et al., 2015; Chen and Wang, 2017; Zhao et al., 2019), acoustic waves (Wang et al., 2007; Cha et al., 2010; Yang J. et al., 2014a), solar irradiance (Stephen, 2006; Zheng et al., 2015; Chen et al., 2016b; Dagdeviren et al., 2017), body heat (Niu et al., 2009; Yang et al., 2013b; Zi et al., 2015a; Wang et al., 2019), and biofuels (Zou et al., 2016), are just some examples of the conversion of energy from and around the human body. In 2012, the triboelectric nanogenerator (TENG) was invented as a highly efficient energy harvesting technology from human biomechanical motions (Bai et al., 2013a; Chen et al., 2013; Hou et al., 2013; Zhu et al., 2013a, 2014b; Yang J. et al., 2014b; Cheng et al., 2015b; Jeong et al., 2015; Chen, 2016; Jin et al., 2016; Wang Z. L. et al., 2016). Compared to other energy harvesting approaches, TENG has several advantages: light weight, low cost, flexible structures, extensive material selection, and great efficiency at low operating frequencies, all of which make TENGs one of the mainstream power supplies for self-powered devices (Jing et al., 2014; Yang W. et al., 2014a,b; Kuang et al., 2015; Lin et al., 2016; Liu R. et al., 2018; Chu et al., 2020; Pu et al., 2020a). TENGs is feasible for driving various electronic devices, ranging from light-emitting diodes (LEDs) (Yang et al., 2013d; Lin et al., 2014; Chun et al., 2015; Kanik et al., 2015; Mao et al., 2015; Wu et al., 2016) to cell phones (Wang et al., 2012; Zhu et al., 2014c) and from a large number of bio-sensors (Fan et al., 2015; Wen et al., 2015; Cai et al., 2018; Su et al., 2018, 2020a,b; Davoodi et al., 2020; Meng et al., 2020) to pacemakers (Zheng Q. et al., 2014). This shows showing their remarkable compatibility with a wide range of application in different settings, displaying that plentiful possibilities are remaining to be further explored (Wang, 2014; Hinchet et al., 2015; Wang S. et al., 2015; Zhang et al., 2015; Zhu et al., 2015; Zhang B. et al., 2016; Pu et al., 2020b).

Note that the triboelectric effect is a well-known phenomenon, in which two surfaces, having different triboelectric properties, become electrically charged during physical contact (Mizes et al., 1990; Liu and Bard, 2009). The principle of TENG is based on the coupling effect of contact electrification and electrostatic induction (Yang et al., 2013c; Su et al., 2014b; Wu et al., 2015; Li Z. et al., 2016; Zhang L. et al., 2016). The static polarized charges, resulting from the contact between the two friction surfaces with different charge affinities, are generated on the friction surfaces and cause different surface potentials, thereby bringing about inductive charges among the two attached electrodes (Su et al., 2014a; Zhu et al., 2014a; Chen et al., 2015a,b). Then the inductive electrons are driven to flow between two electrodes via an external circuit to fulfill the conversion process from mechanical energy to electricity (Niu et al., 2013a,b, 2014a; Chen et al., 2015c; Niu and Wang, 2015; Zi et al., 2015b). The output performance of TENGs is determined by the triboelectric charge density on the triboelectric material surfaces (Dharmasena et al., 2018). Thus, increasing the triboelectric charge density is the most fundamental strategy for building high-performance TENGs. Considerable efforts have been made to increase the triboelectric charge density of TENGs, including proper triboelectric materials selection (Zenkiewicz et al., 2015; Zhao et al., 2015; Kim et al., 2017; Lee et al., 2018), advanced device structural design (Bai et al., 2013b; Lin L. et al., 2013; Wang et al., 2013; Yang et al., 2013a; Zhang H. et al., 2014; Deng et al., 2020), and triboelectric materials surface physical/chemical modifications (Lin et al., 2009; Lin Z. H. et al., 2013; Niu et al., 2014b; Jing and Kar-Narayan, 2018; Zhou Y. et al., 2020). The surface physical modification is primarily realized via material morphological manipulation. Namely, increasing the effective friction area through incorporating surface micro-/nano-structures (Jeong et al., 2014; Kim et al., 2015; Feng et al., 2016; Wang et al., 2017), such as nanowires (Zheng et al., 2014; Lin et al., 2015), nanoparticles (Zhu et al., 2013b) and other nanoscale patterns (Zhang et al., 2013; Lee et al., 2014; Choi et al., 2015; Dudem et al., 2015). Furthermore, manipulating the surface chemistry of the friction layers through chemical modifications and consequent changes in surface potentials will enlarge the polarity between the two friction surfaces therefore contributing to the high-performance of TENGs (Wang S. et al., 2016).

**Figure 1:**
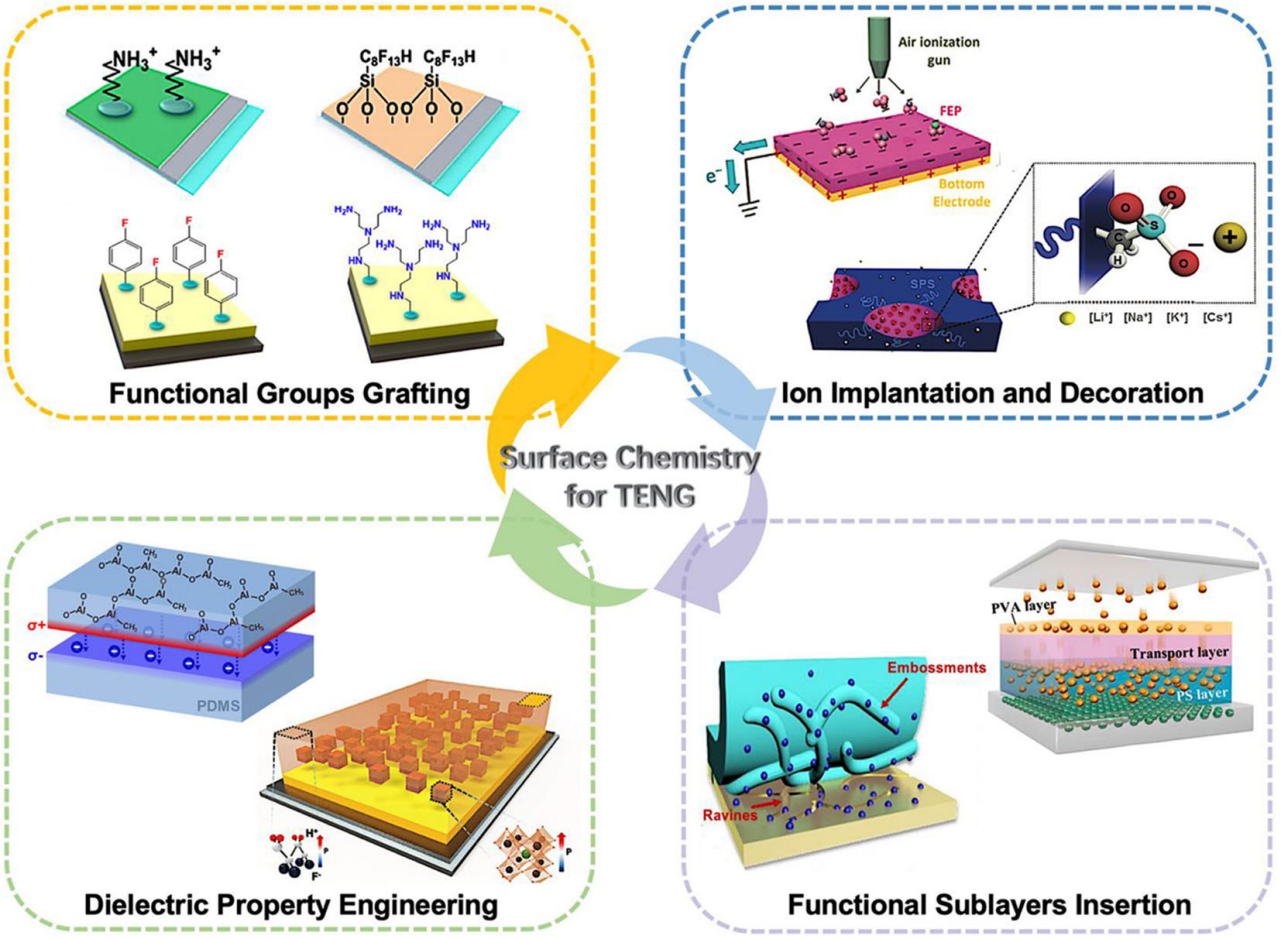
Surface chemistry for high-performance triboelectric nanogenerators. Reprinted with permission from Shin et al. (2017). Copyright 2017 American Chemical Society. Reprinted with permission from Shin and Kwon (2015). Copyright 2015 American Chemical Society. Reprinted with permission from Wang et al. (2014). Copyright 2014 Wiley-VCH. Reprinted with permission from Park et al. (2017). Copyright 2017 WILEY-VCH. Reprinted with permission from Yu et al. (2015). Copyright 2015 Wiley-VCH. Reprinted with permission from Seung et al. (2017). Copyright 2017 Wiley-VCH. Reprinted with permission from Lai et al. (2018). Copyright 2018 American Chemical Society. Reprinted with permission from Cui et al. (2018). Copyright 2018 American Chemical Society.

This review systematically reports the current advances in surface chemistry for high-performance TENGs. As shown in Figure 1, the chemical modification methods can be summarized and classified into four categories: functional groups grafting, ion implantation and decoration, dielectric property engineering, and functional sublayers insertion. In addition, this review provides a critical analysis of surface chemistry for TENG and insights into remaining challenges and future directions. With worldwide efforts in innovations in chemistry and materials elaborated in this review, the frontiers of high-performance TENGs will be pushed forward, which could offer the era of Internet of Things a compelling pervasive energy solution.

## Functional Groups Grafting

Functional groups grafting is a straightforward and efficient method to fabricate high-performance TENG with finely tunable triboelectric properties. By simply introducing electron-accepting and electron-donating groups onto triboelectric material surfaces, functional group grafting outperforms complicated bulk engineering. It indicates that TENGs can be built with a wider range of polymeric surfaces even if they are originally inefficient for triboelectric energy harvesting. This indication unlocks the limitations of triboelectric materials choice for designing high-performance TENGs. In principle, surface functional groups grafting is achieved by chemically grafting target element-containing groups, such as –CF_3_ and –NH_2_ groups, generally through either solution reactions or vapor treatments. By introducing functional groups with high tendency to gain or loss electrons onto the triboelectric surfaces, the negative or positive potential of the surface will be increased and manipulated. This manipulation contributes to a greater transfer charge density during the cyclic contact-and-separate movement between two triboelectric materials and therefore improves the output performance of TENGs. Moreover, applying some surface treatments for functional groups grafting such as the plasma process, can chemically and morphologically modify surfaces simultaneously (Zhang X. et al., 2014; Cheng et al., 2015a; Li et al., 2015), which can further improve the output performance of TENGs. In this section, methods concerning functional groups grafting on the triboelectric material surfaces are introduced, including their enhancement mechanism toward TENG performance.

### Self-Assembled Monolayer

The self-assembled monolayer (SAM) method, wherein the chemical adsorption of an active surfactant on a solid surface is used to realize well-ordered molecular assembly can be easily performed and widely used on various kinds of surfaces (Ulman, 1996; Song et al., 2015). Using the SAM method in surface functionalization of TENGs, the target functional groups can be anchored onto the material surfaces through forming strong chemical bonds, effectively altering the surface potential of contact materials. Unlike simply functionalized surface via one-step solution reactions or vapor treatments (Yao et al., 2017), this method is effective not only on noble-metal substrates but also on insulating substrates, such as SiO_2_ and Kapton. Moreover, the uniform and well-ordered SAMs in nanoscale effectively controlled surface defects and reduced the adsorption of additional adsorbates in the air (Rimola et al., 2013). In this way, the SAM method can extensively expand the scope of triboelectric material choices and enhance the performance of TENGs.

**Figure 2:**
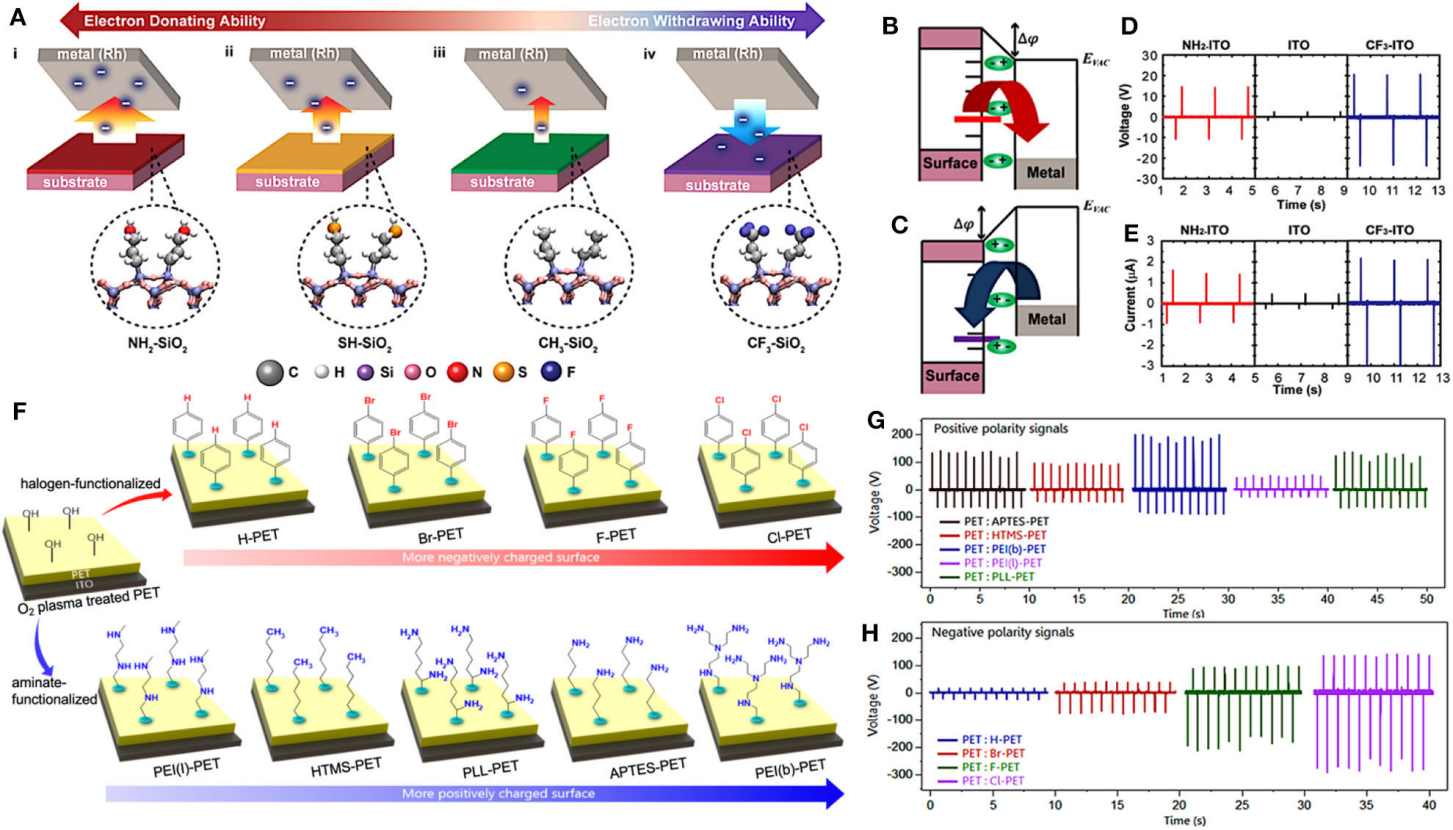
Schematic representations of SAM-modified triboelectric materials and their output performance before and after functionalization. **(A)** Schematic diagram showing the propensity of the triboelectrification on (i) a strongly electron-donating layer (–NH_2_), (ii) a moderate electron-donating layer (–SH), (iii) a neutral layer (–CH_3_), and (iv) an electron-accepting layer (–CF_3_). **(B)** Energy band diagrams of substrates modified with an electron-donating layer and **(C)** with an electron-accepting layer and that of the metal. **(D)** Open-circuit voltage and **(E)** short-circuit current of the triboelectric device composed of PI and SAM-modified ITO. Reprinted with permission from Byun et al. (2016). Copyright 2016 American Chemical Society. **(F)** Schematic illustrations of surface functionalized polyethylene terephthalate (PET) substrates with various halogen-containing and aminated molecules. **(G)** Output voltage generated by cyclic contacts between bare PET:aminated-PET pairs and **(H)** bare PET:halogenated-PET pairs. Reprinted with permission from Shin et al. (2017). Copyright 2017 American Chemical Society.

Byun et al. systematically modulated the triboelectric polarities of SiO_2_ layers by functionalizing the surfaces with various electron-donating functional groups, including –NH_2_, –SH, a neutral group –CH_3_, and an electron-accepting functional group, –CF_3_ (Byun et al., 2016) Figure 2A shows a schematic diagram depicting the triboelectrification of these various SAM- modified SiO_2_ layers. The modification process started with treating the substrates, a wafer with a 100 nm-thick SiO_2_ layer, by ultraviolet/ ozone plasma for 10 min to prepare for functional group anchoring. After that, in the lone case of –NH_2_, the substrate was immersed in a 1% (v/v) (3-aminopropyl) triethoxysilane (APTES)/ethanol solution for 1 h. The other three functional groups, –SH, –CH_3_, and –CF_3_, were all formed by the chemical vapor deposition method (Hozumi et al., 1999; Hayashi et al., 2002). During these processes, target functional groups were anchored on the surface of triboelectric materials, which altered the conventional triboelectric series of original substrates, leading to a higher tendency of triboelectric surfaces to lose or gain electrons. Figures 2B,C show energy band diagrams of surface modified with an electron-donating and an electron-accepting functional groups. Among these functional groups, the negative and positive surface dipoles introduced by the strong electron-donating –NH_2_ groups and electron-accepting –CF_3_ groups can significantly decrease or increase the surface potential. The relative position of the as-functionalized surfaces and the counter materials in energy level affects the direction of electrons flow. It reveals that by modulating the surfaces with various electron-donating and -withdrawing functional groups, the polarity and the amount of triboelectric charge on the material surfaces can be well-controlled. In principle, TENG with as-SAMs-modified surfaces should deliver significant electric output considering the enlarged difference of friction surfaces potential. Figures 2D,E compare the open-circuit voltage and the short-circuit current of TENGs consisting of the SAMs-modified indium tin oxide (ITO) and polyimide (PI). With SAMs-modified surfaces: NH_2_-ITO and CF_3_-ITO, the output performance of as-fabricated TENGs have remarkable enlargement in both *V*_*oc*_ and *I*_*sc*_, compared to that of using a bare ITO surface.

Similar SAMs functionalization was applied to conventional TENG materials, such as polyethylene terephthalate (PET) substrates through a series of halogens and amines. Shin et al. obtained a wide spectrum of tunable triboelectric polarity through chemical surface functionalization with the halogen-containing molecules and the aminated molecules (Shin et al., 2017). Before anchoring functional groups to the PET surfaces, the PET substrates were first treated by oxygen plasma to form hydroxyl groups (–OH) on the surfaces, as shown in Figure 2F. Here, the –OH groups played a crucial role as strong chemical bonds between the surfaces of PET substrates and the target functional molecules. The surface was then functionalized with electron-accepting elements, halogens (Br, F, and Cl)-terminated phenyl derivatives and several aminated molecules to induce triboelectrically negative or positive property on PET substrates. Figures 2G,H show the output voltages of TENGs with bare PET:aminated-PET contact pairs and bare PET:halogenated-PET contact pairs, respectively. The result shows a significant variation in the output performance. The TENGs with the aminated-PET surfaces generated strong positive polarity signals, while negative polarity signals were generated from the TENGs with halogenated-surfaces in contrast. Among them, the PEI(b)-PET:PET and Cl-PET:PET contact pairs generated the maximum output voltage, exceeding 300 and 200 V, respectively.

**Figure 3:**
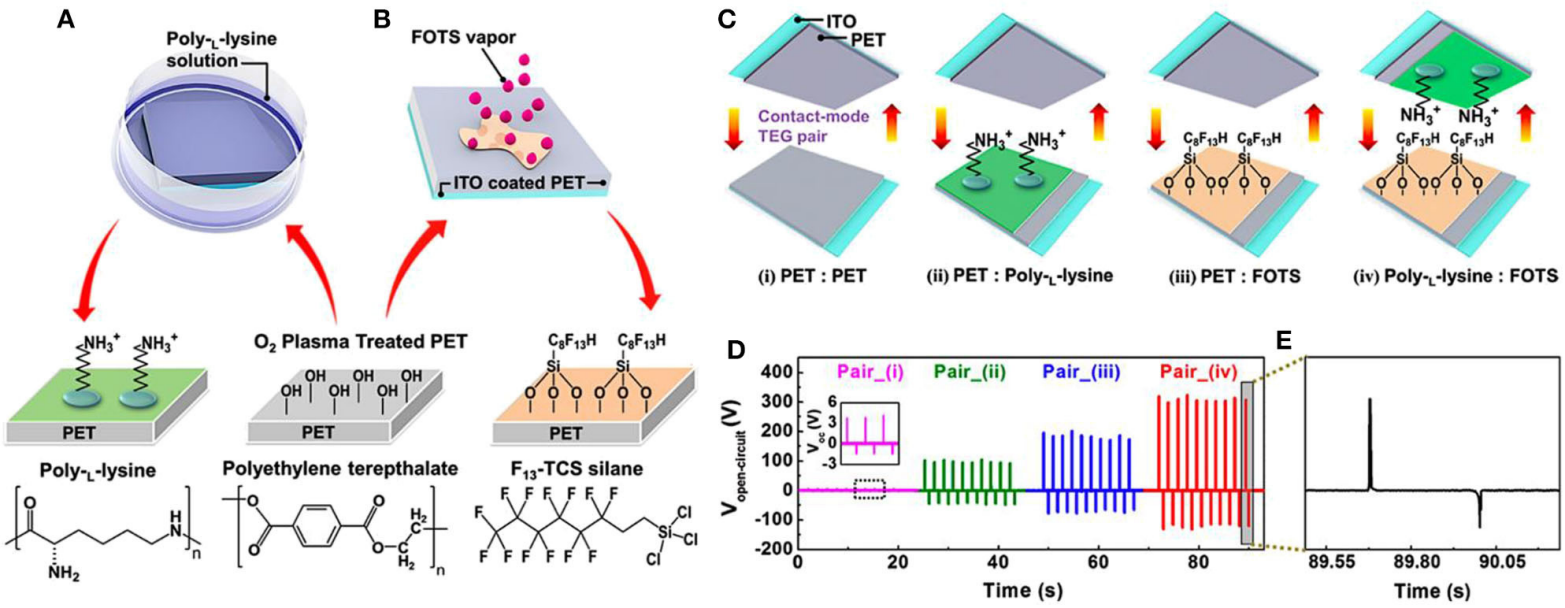
Schematic illustration of modification processes and output performance of TENGs with functionalized-surfaces. **(A)** The surface modification process by dip-coating and **(B)** self-assembling deposition. **(C)** Schematic of TENGs fabricated with different contact pairs. **(D)** Open-circuit voltage of corresponding TENGs. **(E)** Local enlarged peaks of the open-circuit voltage. Reprinted with permission from Shin and Kwon (2015). Copyright 2015 American Chemical Society.

For high-performance TENGs designation, SAM method, cooperating with other efficient surface functionalization approaches, is applied flexibly to modulate the triboelectric polarities of both triboelectric materials. Shin et al. engineered the surfaces of triboelectric materials with a negatively charged –CF_3_ group via self-assembling deposition and with positively charged –NH_3_ group through dip-coating (Shin and Kwon, 2015). Figures 3A,B display the surface modification process, where firstly PET substrates were treated by oxygen plasma for 100 s to form reactive –OH groups strongly binding on their surfaces. Subsequently, one plasma-treated PET film was dipped in the poly-l-lysine (PLL) solution for 5 min, while the other side was exposed in trichloro(1H,1H,2H,2H-perfluorooctyl) silane FOTS vapor environment at 95°C for 1 h. During this processing step, target molecules (PLL and FOTS) are anchored onto the surfaces of PETs through covalent bonding with –OH groups. Consequently, the PPL-coated PET (P-PET) surface is positively charged by –NH_3_ groups and the self-assembling chemical vapor treated PET (F-PET) surface is negatively charged by –CF_3_ groups. Figure 3C displays the schematic of TENGs fabricated with various triboelectric material pairs: (i) bare PET:bare PET, (ii) bare PET:P-PET, (iii) bare PET:F-PET, (iv) P-PET:F-PET. Furthermore, the comparisons of their open-circuit voltages are shown in Figures 3D,E. Among these TENGs, the generated output voltages increase from ~4 V in the device with PET:PET contact pair to ~330 V with P-PET:F-PET pair. Thus, the output performance of TENGs can be greatly enhanced by modulating both friction surfaces and corresponding triboelectric polarities, implying the outstanding effectiveness of these functional group modification methods. In addition, the surface of the functionalized PET demonstrates superior stability, which is attributed to its tight chemical bonds with surface functional groups.

### Ultraviolet-Ozone Irradiation

**Figure 4:**
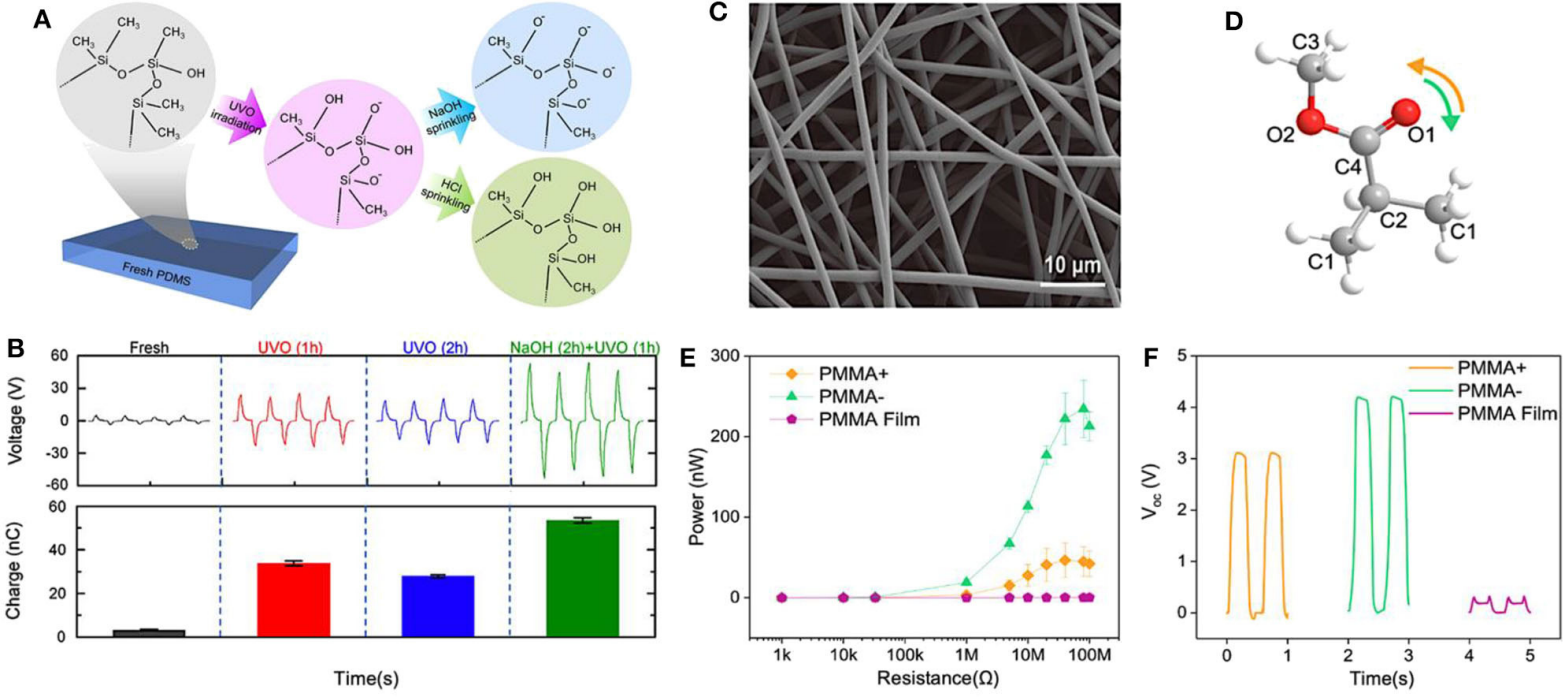
Schematic diagrams and output performance of functionalized triboelectric materials surfaces via UVO irradiation or electrospinnig. **(A)** Schematic diagram about suggested mechanism of chemical elements and bonds of PDMS surface after UVO irradiation and NaOH treatment. **(B)** Comparison of the open-circuit voltage and induced triboelectric surface charge of surface-treated TENGs based on fresh PDMS, UVO-irradiated PDMS for 1 h [UVO (1 h)] and 2 h [UVO (2 h)], and NaOH-treated PDMS for 2 h after UVO irradiation for 1 h [NaOH (2 h)+UVO (1 h)]. Reprinted with permission from Yun et al. (2015). Copyright 2015 Elsevier. **(C)** SEM image of electrospun PMMA fibers. **(D)** Schematic structure of a single unit of PMMA polymer chain. **(E)** Output power and **(F)** open-circuit voltage of electrospun PMMA-based TENGs. Reprinted with permission from Busolo et al. (2018). Copyright 2018 Elsevier.

In addition to the SAM method, Ultraviolet-ozone (UVO) irradiation is another facile method to enhance the triboelectric charge of triboelectric material surfaces via tailoring the chemical functional groups (Hoek et al., 2010; Fan et al., 2014). Unlike the SAM method, UVO irradiation creates desired functional groups by substituting the existing chemical elements and bonds on the surface with target elements and new bonds, instead of directly introducing new chemical functional groups onto the material surfaces. Yun et al. increased the triboelectric surface charge of polydimethylsiloxane (PDMS) via the UVO irradiation method. After that, by simply sprinkling NaOH or HCl solution onto the UVO-irradiated PDMS surfaces, the triboelectric surface charge was further changed significantly (Yun et al., 2015). As shown in Figure 4A, the fresh PDMS surface consists of mainly non-polar Si-CH_3_ bonds (Cole et al., 2011). During UVO irradiation, the previous Si–CH_3_ bonds are broken and converted to polar Si–O, Si–OH, and Si–COOH bonds, obtaining a mildly base and polar surface. Further NaOH treatment by simply sprinkling NaOH solution onto the UVO-treated PDMS surface results in an additional increase of the amount of Si–O bonds at the expense of Si–CH_3_ bonds. When adopting the HCl treatment, on the other hand, the Si–O bonds are changed to Si–OH bonds (Lai et al., 2012). Due to a large amount of polar Si–O bonds on the UVO- and NaOH-treated PDMS surface, a greater triboelectric charge was generated, leading to higher output performance of as-treated PDMS based TENGs. As for the UVO- and HCl-treated PDMS surface, the result showed relatively little triboelectric charge and lower output performance of as-fabricated TENGs. Figure 4B compares the output performance of fresh and surface-treated PDMS-based TENGs. After surface treatment with NaOH for 2 h and UVO irradiation for 1 h, the open-circuit voltage and short-circuit current exhibited an almost 15-fold enhancement than those of fresh PDMS-based TENGs reaching 49.3 V and 1.2 μA, respectively. Note that, the output voltage and current of TENGs can be improved by simple UVO treatment for 1 h, shown in Figure 4B with red lines. However, when increasing the exposure time to 2 h, the output voltage and current showed no further enhancement, as shown with blue lines. An in-depth comparison of triboelectric surface charge induced from fresh and surface-treated PDMS-based TENGs is displayed too. The triboelectric charges of UVO (1 h) and NaOH (2 h)+UVO (1 h) treated PDMS were enhanced significantly, with ~33.9 and 53.5 nC respectively, when compared to that of fresh PDMS with merely 3.17 nC. While increasing the UVO treatment time to 2 h, the surface-induced charge decreases. It implies that NaOH solution treatment after UVO irradiation is an easy-achieved and efficient method to enhance the output performance of TENGs, but controlling the UVO irradiation time is critical to optimize efficiency.

### Electrospinning

The electrospinning technique is a one-step method to tailor the surface potential of triboelectric materials and consequently improve TENGs triboelectric output performance. Electrospinning is applied in the materials fabrication process; a high voltage is applied between the nozzle of a syringe filled with a polymer solution and the grounded substrate to produce solid fibers on the substrate. In this case, through applying a positive or negative voltage to the nozzle during the electrospinning process, charges of equivalent polarity are generated at the liquid jet–air interface, altering the surface chemical properties of materials (Stachewicz et al., 2012). Busolo et al. altered the surface chemistry of polymethylmethacrylate (PMMA) fibers via electrospinning and substantially improved triboelectric output performance of electrospun PMMA based TENGs (Busolo et al., 2018). The PMMA fibers altered by polarized charges were successfully fabricated as scanning electron microscopy (SEM) images shown in Figure 4C. Positive (PMMA^+^) and negative (PMMA^−^) charges were generated by applying a positive and negative voltage to the nozzle, respectively, to modify the surface chemistry. The changes in surface chemistry were analyzed by using X-ray photoelectron spectroscopy and the result data indicated that the variation of the surface potential properties between PMMA^+^ and PMMA^−^ fibers were directly related to the distinct contents of chemical bonds in the units of PMMA polymer chain, as shown in Figure 4D. In the case of positive voltage polarity applied during electrospinning, more oxygen-containing groups were present at the O1 and O2 regions, whereas, in the case of negative voltage polarity applied, the units contained more C–C chemical bonds. Thus, due to the high electronegativity of oxygen, PMMA^+^ fibers exhibited a lower surface potential when compared to PMMA^−^ fibers. The changes in surface potential were correlated with variations of charge transfer affinity and triboelectric performance. To investigate the output performance of as-fabricated TENGs, PMMA^+^ and PMMA^−^ fibers were deposited onto an aluminum foil by electrospinning. The modified aluminum foil served as one of the triboelectric material. Via periodically stimulating the contact-and-separate movement with the counter-electrode (copper substrate), the output power and voltage of PMMA^+^ fiber, PMMA^−^ fiber, and pure PMMA film are obtained and compared in Figures 4E,F. Contact pairs based on PMMA^+^ and PMMA^−^ fibers significantly outperformed PMMA film-based TENGs in both output power and open-circuit voltage. The PMMA^−^ fibers produced a maximum power output of 234.4 nW at a load resistance of 80 MΩ and obtaining a nearly 10-fold enhancement in voltage. These results indicated that the electrospinning technique can be adopted to effectively manipulate the surface potential of fibers by alternating the polarity of applied voltage in a simple manufacturing process; therefore, substantially improving the triboelectric output.

In conclusion, functional groups grafting is a straightforward, cost-efficient and easy-to-implement method to manipulate the surface chemical properties, by breaking and forming new chemical bonds on triboelectric materials' surfaces. New functional groups with strong electron-donating or electron-accepting ability are anchored onto the materials surface, giving rise to drastic performance enhancement of TENGs. However, since the functional groups grafting is just taken place on the surfaces rather than deep into the bulk of tribo-materials, the results may lose its effectiveness if the surface is polished or worn out during the friction.

## Ion Implantation and Decoration

Ion implantation and decoration, is the method that directly adds ions or single-polarity charged particles on or inside the triboelectric materials of interest. This can be an efficient way to increase the charge density of the triboelectric surfaces and thus enhance the output performance of TENGs. Such methods, including ion injection and irradiation, usually are achieved with the assistance of special instruments, such as, an air-ionization gun. Additionally, the modifications do not only take place on the material surfaces, but also affect both surface and bulk regions. This indicates that the results can be maintained for several months or even longer (Wang et al., 2014), implying that the as-fabricated high-performance TENGs will have better output stability.

### Ion Injection

**Figure 5:**
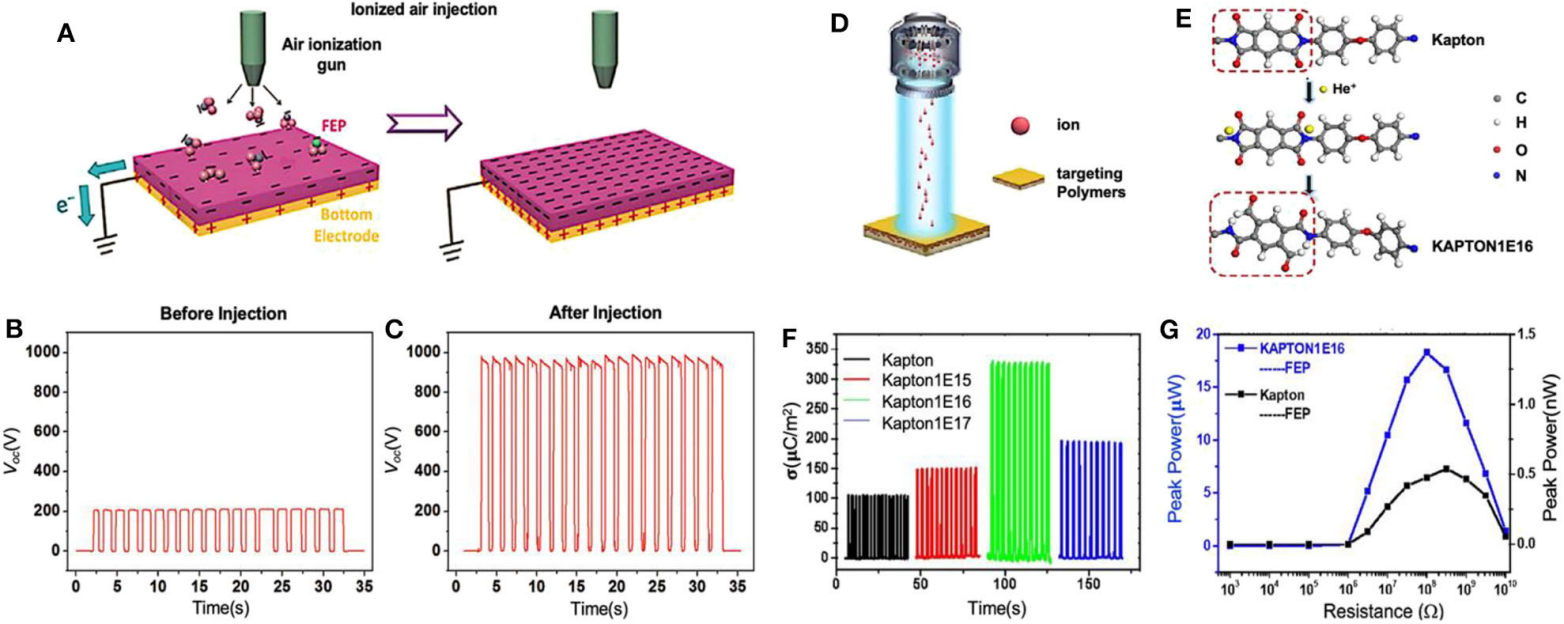
Basic modification processes and output enhancement of triboelectric materials surfaces via ion injection and ion irradiation. **(A)** Schematic diagram of the ionized ion injection applying onto FEP surface with air ionization gun. **(B,C)** Open-circuit voltage of TENGs based on FEP films **(B)** before and **(C)** after the ion injection process. Reprinted with permission from Wang et al. (2014). Copyright 2014 Wiley-VCH. **(D)** Schematic diagram of ion irradiation simulation. **(E)** Schematic diagram of chemical structure changes of Kapton. **(F)** Transferred charge density and **(G)** output power of different ion concentration irradiated Kapton contracting with fluorinated ethylene propylene (FEP) films. Reprinted with permission from Li et al. (2020). Copyright 2019 Royal Society of Chemistry.

The ion injection technique, which is applicable to various triboelectric polymers, is an effective and the most commonly adopted way to introduce surface charges for enhancing TENGs' output performance. The ions with negative or positive polarities are generated and subsequently implanted onto the material surfaces by triggering the discharge of air in the air-ionization gun. Wang et al. adopted this method to create negative charges ${\text{CO}}_{3}^{-}$, ${\text{NO}}_{3}^{-}$, ${\text{NO}}_{2}^{-}$, ${\text{O}}_{3}^{-}$, and ${\text{O}}_{2}^{-}$, and injected them onto the fluorinated ethylene propylene (FEP) surface (Baytekin et al., 2011). In order to achieve the maximum surface charge density for high-performance TENG, the bottom electrode attached to the back of the FEP film was grounded, as shown in Figure 5A. In this way, the negative charges introduced by ion injection will drive equal quantities of electrons away from the bottom electron to the ground, leaving positive charges in the electrode. The grounded connection of the bottom electron will lead to a higher surface charge density when compared with the electrode-free FEP layer, because FEP has a much larger dielectric property than air. By repeating this procedure multiple times, the negative charge density on the material surface can be controlled to reach any desired level. The large amount of the negative or positive polarities charges distributed on the surface of the contact materials through the ion injection can lead to a strong driving force for the high-output voltage and current. In this work the results showed a five-fold enhancement of the surface charge density is obtained by the ion injection. Figures 5B,C compare the *V*_*oc*_ of TENGs fabricated with FEP films before and after ion injection, respectively. The FEP film is assembled with an Al layer, as a pair of triboelectric materials, to form a contact-mode TENG. Before injection, as-fabricated TENG can only produce a *V*_*oc*_ of ~200 V, because its surface charges are generated merely by the triboelectrification. After the injection, which introduced a large number of external charges onto the surface, the *V*_*oc*_ was enhanced to ~1,000 V. Moreover, the output power of the modified-TENGs was elevated by 25 times, which was also proven to be stable over 5 months and 400,000 continuous operating cycles.

### Ion Irradiation

Ion irradiation is a widely used method for many materials such as metals, superconductors and semiconductors, etc., owing to its controllable applied area, adjustable ion dose, and uniform treated results (Dong and Bell, 1999; Awasthi et al., 2010; Kumar et al., 2013; Kumar and Singh, 2015; Wang et al., 2015). Via low-energy ion irradiation, Li et al. proposed a novel surface modification process to effectively modulate the chemical structures of the target polymer, Kapton (Li et al., 2020). Unlike the Ion Injection method mentioned above, which can significantly improve the achievable maximum charge density on the tribo-materials surfaces by electrostatically inducing the same amount of electrons flowing from the bottom electrode to the ground, Ion Irradiation mainly changes the crystal structure of the target materials and consequently changes its surface properties via inducing the breaking and rearrangement of chemical bonds. Figure 5D shows a schematic diagram of ion irradiation simulation, where a low-energy ion beam of 50 keV He was used to irradiate the Kapton surface. Meanwhile, various ion irradiation concentrations are adopted on the polymer, changing from 1 × 10^15^ to 1 × 10^17^ ions/cm^2^ (as-treated Kapton polymers were named Kapton1E15, Kapton1E16, and Kapton1E17, correspondingly). The collisions produced by ion irradiation cause macromolecules in the polymer to reach a chemical bond energy barrier and finally break. After that, the implanted ions combine onto polymer surfaces by forming new chemical bonds. Take the ion irradiation process of Kapton1E16 as an example, shown in Figure 5E. The original C-N bonds were broken and C-H and N-H bonds were formed instead after low-energy He ion implantation. In this way, the -NHCOR bond, which is a very strong electron donating group when conjugated with a benzene ring, was established on the surface of the polymer. This is circled out with the red dotted line in the Kapton1E16 molecular formula. As a result, Kapton film modified by ion irradiation shows higher surface charge density, excellent stability, and ultrahigh electron-donating capability. Therefore, this provides a good demonstration for manipulating the output performance of TENGs based on controllable chemical structure change. The transferred charge density of different ion concentration irradiated Kapton contracting with fluorinated ethylene propylene (FEP) films were shown in Figure 5F. Among these four samples, Kapton1E16 film achieved the maximum transferred charge density with 332 μC/m^2^. Moreover, the output power of Kapton1E16 and pre-irradiated Kapton polymer are compared in Figure 5G, implying a significant enhancement in maximum peak power after ion irradiation treatment, from 0.5 nW to 18 μW. To further verify the stability of Kapton1E16, the as-fabricated TENG was tested for more than 60 days without output degradation, which proved that the chemical modification of Kapton film induced by ion irradiation is quite stable and effective.

### Ion Absorption

**Figure 6:**
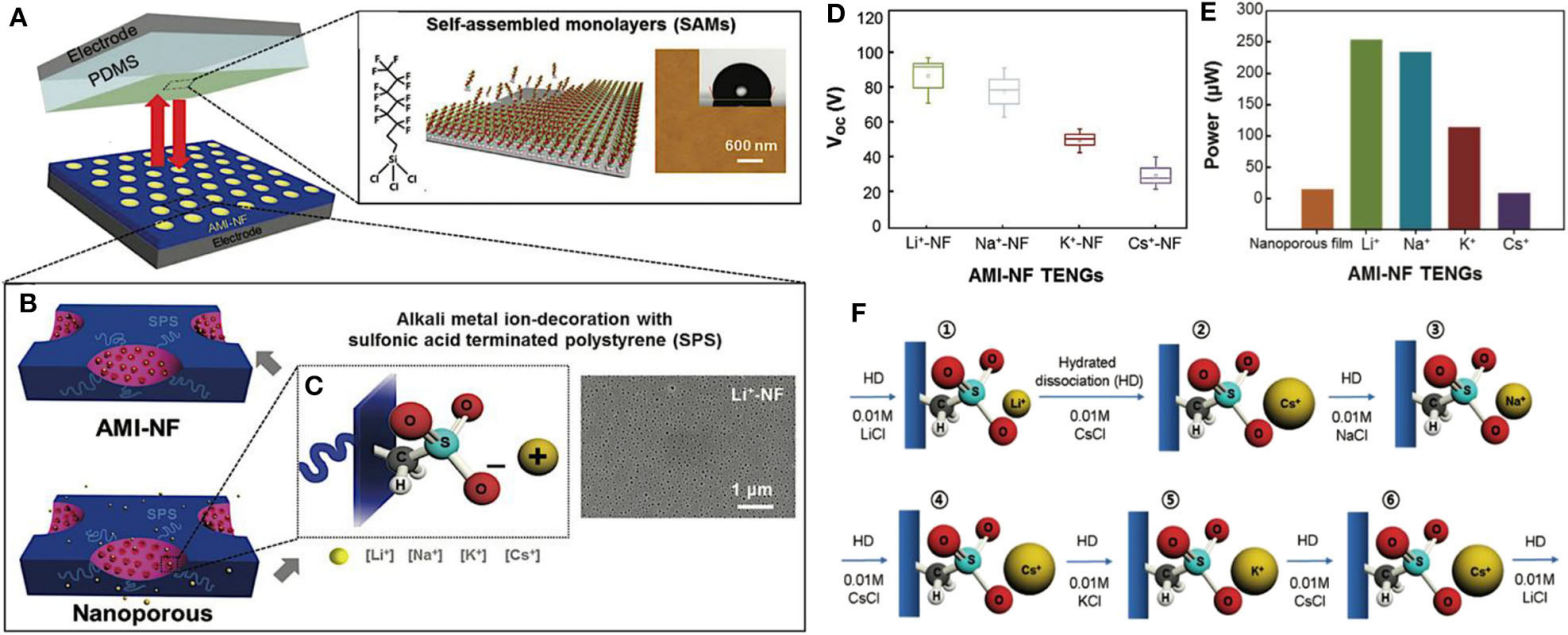
Schematic diagrams and output performance of TENGs with ion-decorated triboelectric materials. **(A)** Schematic diagram of the structure of an AMI-NF TENG. **(B)** Schematic diagram of nanoporous structure on the surface of AMI-NF. **(C)** Partial enlarged schematics of alkali metal ion (Li^+^, Na^+^, K^+^, and Cs^+^) association with sulfonate (${\text{SO}}_{3}^{-}$) functional groups on the surface of the nanopores. **(D)** Average *V*_*oc*_ and **(E)** averaged maximum power of AMI-NF with various alkali metal ions. **(F)** Schematics of sequential alkali metal ion exchange on a nanoporous surface: ① Li^+^→ ② Cs^+^→ ③ Na^+^→ ④ Cs^+^→ ⑤K^+^→ ⑥ Cs^+^→ ① Li^+^. Reprinted with permission from Park et al. (2017). Copyright 2017 WILEY-VCH.

Alkali Metal ions can also be used to modify the surface chemistry of some types of triboelectric materials by simple solution-processed alkali metal ion absorption. As shown in Figure 6A, Li et al. chose SAM-modified PDMS and the alkali-metal-ion-decorated nanoporous film (AMI-NF) as a pair of triboelectric materials for TENGs fabrication (Park et al., 2017). The modification process started from the preparation of the supramolecular-assembled nanoporous film, where 6:4 w/w blends of sulfonic-acid-terminated poly(styrene) (SPS) and poly(2-vinylpyridine) (P2VP) were used as a precursor. The subsequent removal of P2VP from the precursor, where the pyridine nitrogen atoms of P2VP had been ionically linked to the sulfonic acid groups of SPS by acid-base interaction, resulted in a nanoporous surface enriched with sulfonic acids (${\text{SO}}_{3}^{-}$ groups) that could be used for binding alkali metal ions, as shown in the diagram in Figure 6B. On the other side, PDMS film was modified via vapor deposition to form a layer of uniform FOTS on the surface. Following the procedures, we introduced before in the section of the SAM method, as-modified PDMS film obtains a fluorinated surface that has a strong tendency to attract electrons. Next, the ions chosen in the work to modify the nanoporous friction film were alkali metal ions, including Li^+^, Na^+^, K^+^, and Cs^+^. By simply immersing the nanoporous film into an aqueous solution containing these alkali metal ions, Li^+^, Na^+^, K^+^, and Cs^+^ ions can be absorbed into the nanopores of the materials and selectively bind with ${\text{SO}}_{3}^{-}$ groups, as schematically shown in Figure 6C. Since the electrostatic field built by cation field can lead to the polarization effect of the sulfonate group (Kujawski et al., 1992; Salis and Ninham, 2014), the electron transfer ability from AMI-NF to PDMS increases upon the tribo-contact after metal ions decoration, allowing us to fabricate alkali-metal-ion-decorated TENGs with better output performance and mechanical properties. Figures 6D,E show the average open-circuit voltage and the maximum power of AMI-NFs modified with various alkali metal ions. The average *V*_*oc*_ values of AMI-NF TENGs with Li+, Na+, K+, and Cs+ ions were −90, −79, −49.2, and −27.4 V, respectively. For comparison, the output power of nanoporous film without alkali-metal-ions absorption was examined with AMI-NF TENGs, showing that the power generated from the bare nanoporous film was much poorer than that of the Li^+^, Na^+^, K^+^-NF TENGs. The averaged maximum power of Li^+^, Na^+^, K^+^-NF TENGs was 256.5, 238.4, 115.7, and 11.5 μW, respectively. The value of 256.5 μW in the case of Li + -NF is notably high and 20 times higher than that of the bare nanoporous film with a value of only 12.8 μW. The lowest output performance of bare nanoporous film attributes to the weakest polarization without alkali metal ions effect. Therefore, the friction film obtains the least effective electron transfer ability when in contact with the PDMS friction film, resulting in a TENG with the lowest output performance. Furthermore, in the stability test of as-modified TENGs, the device with a size of 2 × 3 cm was mechanically robust and could be operated without performance degradation under repetitive contacts (1.25 Hz, 5 N) for more than 50,000 cycles. The results suggest that chemical modification of the triboelectric material surface with alkali metal salts is crucial for high-performance TENGs with relatively long effective duration.

Interestingly, as-fabricated AMI-NF TENGs offers a convenient means to switch the triboelectric properties via a simple solution treatment, since alkali metal ions bound with ${\text{SO}}_{3}^{-}$ groups on the nanoporous surface can be readily exchanged from one to another, as shown in Figure 6F. Each step (from to ) shown in the diagram involves the hydrated dissociation (HD) of the alkali metal ions from the ${\text{SO}}_{3}^{-}$ groups with AMI-Cl solution. In this way, a controllable *V*_*oc*_ and output power, ranging from −90 to −27.4 V and 11.5–256.5 μW, respectively, were obtained simply by a conventional ion exchange process in a reversible manner. Thereby, wide-range tuning of triboelectric output performance can be achieved.

In summary, the ion injection and decoration methods provide effective approaches for improving the TENG's output performance. Since modification took place upon both the surface and near-surface regions, the enhanced output performance can last stably at least for months and will not suffer from the polish of the surfaces. Thus, it is the most commonly adopted method of materials surface modification for improving TENGs' output performance by far. Nevertheless, the fabrication processes are relatively complicated and the instrument used during the process adds extra cost, which may bring obstacles for further scaling up.

## Dielectric Property Engineering

Controlling the dielectric property of triboelectric materials can also effectively enhance the output performance of TENGs. The underlying mechanism of the dielectric property engineering shares similarities with the above-mentioned ion implantation and decoration methods. Both of them manipulate the output performance of TENGs by controlling the surface charge density of triboelectric materials or introducing extra charged ions or molecules. Compared to other chemical modification methods, the dielectric property engineering improves the triboelectric materials' binding force and holding capacity of electrons. This method achieves such characteristic enhancements by doping high dielectric nanomaterials into its bulk region. This bulk modification, which manipulates the chemical properties of the material itself instead of just modifying the outermost surface, will then be reflected on the changes in surfaces' charge density. Certainly, this approach also contributes to developing high-performance TENGs with good resistance to the surface wearing issue. Moreover, dielectric property modification is versatile in tailoring a broad range of triboelectric materials since a variety of metalorganic molecules possess high permittivity in most polymer chains (George, 2010; Jin et al., 2020). With proper selection of dopants and precise control of the dose, dielectric property modification could be a promising approach to improve the output performance of TENGs.

### Sequential Infiltration Synthesis

**Figure 7:**
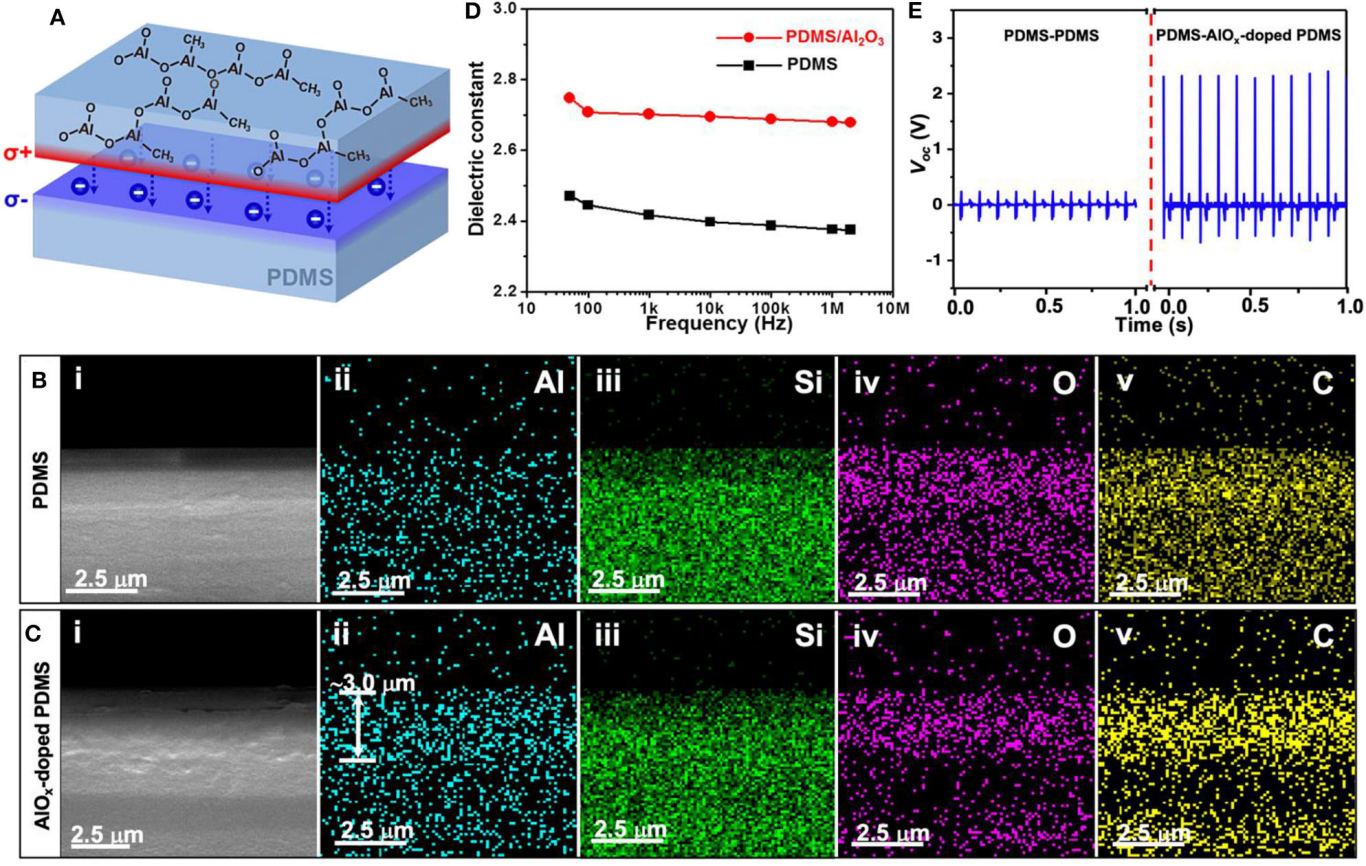
SEM images of SIS-modified triboelectric materials and output performance of as-fabricated TENG. **(A)** Schematic diagram of triboelectrification induced charge redistribution between pristine PDMS and AlO_x_-doped PDMS films upon contact. **(B,C)** Cross-sectional SEM images (i) and corresponding energy dispersive spectroscopy (EDS) mappings of a pristine PDMS film and an AlO_x_-doped PDMS film for Al, Si, O, and C elements (ii–v). **(D)** Dielectric constant of pristine PDMS film and AlO_x_-doped PDMS film. **(E)** *V*_*oc*_ of TENGs based on the PDMS with and without AlO_x_ doping. Reprinted with permission from Yu et al. (2015). Copyright 2015 Wiley-VCH.

Sequential infiltration synthesis (SIS) is a molecular infiltration process on the basis of the atomic layer deposition (ALD) technique (Wilson et al., 2005; Peng et al., 2011; Biswas et al., 2015). The method is applied in the process of polymer fabrication; the larger permittivity of metalorganic ALD precursors allows deep infiltration of inorganic compounds during the ALD process, leading to inorganic-and-organic hybrid materials (Ferguson et al., 2004; Gong et al., 2012a,b; Moghaddam et al., 2013; Padbury and Jur, 2014). Through deep infiltration of some high dielectric inorganic molecules, such as AlO_x_, the SIS technique was expected to be an effective means to tailor the dielectric and electrical properties of the polymer of interest and therefore provide a solution for modifying triboelectric materials in bulk volume. Based on the SIS technique, Yu et al. proposed an internal AlO_x_ doping method of several triboelectric polymers, including PDMS, Kapton and PMMA (Yu et al., 2015). AlO_x_ was selected as the dopant since trimethylaluminum (TMA, ALD precursor of Al_2_O_3_) has desirable permittivity in a number of polymers such as polystyrene (PS), polypropylene (PP), polyethylene (PE), and poly (vinyl chloride) (PVC) and PMMA (Seung et al., 2015). The modification results of the PDMS film serves as a good as simultaneous surface and bulk modification of the PDMS films' dielectric and electrical properties were achieved, the schematic diagram is shown in Figure 7A. The AlO_x_-doped PDMS is capable of storing a greater volume of charges compared with the pristine one. Moreover, the surface potential of the AlO_x_-doped PDMS film was changed due to the injection of electrons from AlO_x_-doped PDMS film to pristine PDMS film upon their friction. This subsequently leads to a positively charged surface in the doped PDMS film and negatively charged surface in the pristine PDMS film. As we know, such a charge redistribution will not happen between two PDMS films without modification, due to their identical surface potential. Therefore, the AlO_x_ doping process via the SIS technique should be able to significantly raise the output performance of TENGs.

As a consequence of the modification, Figures 7B,C show the cross-sectional SEM images and corresponding energy dispersive spectroscopy (EDS) elemental mappings of the pristine PDMS film and the AlO_x_-doped PDMS film after 5-cycle sequential TMA/H_2_O infiltration, respectively. This elemental distribution analysis indicated the successful deep infiltration of molecules in the PDMS film rather than the surface coating of Al_2_O_3_ molecules. From these diagrams, AlO_x_ molecules were found to be capable of penetrating as deep as ~3 μm into PDMS film, since there was a high concentration of ions gathering within this region. This bulk modification attributed to the deep doping method, allows the enhanced performance of TENGs to survive even after polishing off 2 μm-thick polymer materials, leading to a longer duration of effective time. Figure 7D displays the dielectric constant of pristine PDMS film and AlOx-doped PDMS film. The dielectric constant of PDMS films increased from ~2.4 to ~2.7 after the SIS doping technique, implying that AlO_x_-doped PDMS was capable of storing more charges than the pristine one. By controlling the types and doses of dopant and modified triboelectric materials, the SIS doping could also arbitrarily tune the charge attraction or repulsion ability of the polymer, therefore, successfully manipulating the output performance of TENGs, similar to previously discussed modification strategies. The average peak values of the *V*_*oc*_ of TENGs fabricated with the two pristine PDMS pair and AlO_x_-doped PDMS: PDMS pairs are shown in Figure 7E. The value reached 2.3 V in the AlO_x_-doped pair which were comparable to TENGs based on typical triboelectric pairs with distinct electron affinities, such as PDMS-ITO and Teflon-metal pairs (Fan et al., 2012), while the untreated PDMS pair generated only 0.3 V in the same testing condition.

### High Dielectric Nanoparticles Doping

**Figure 8:**
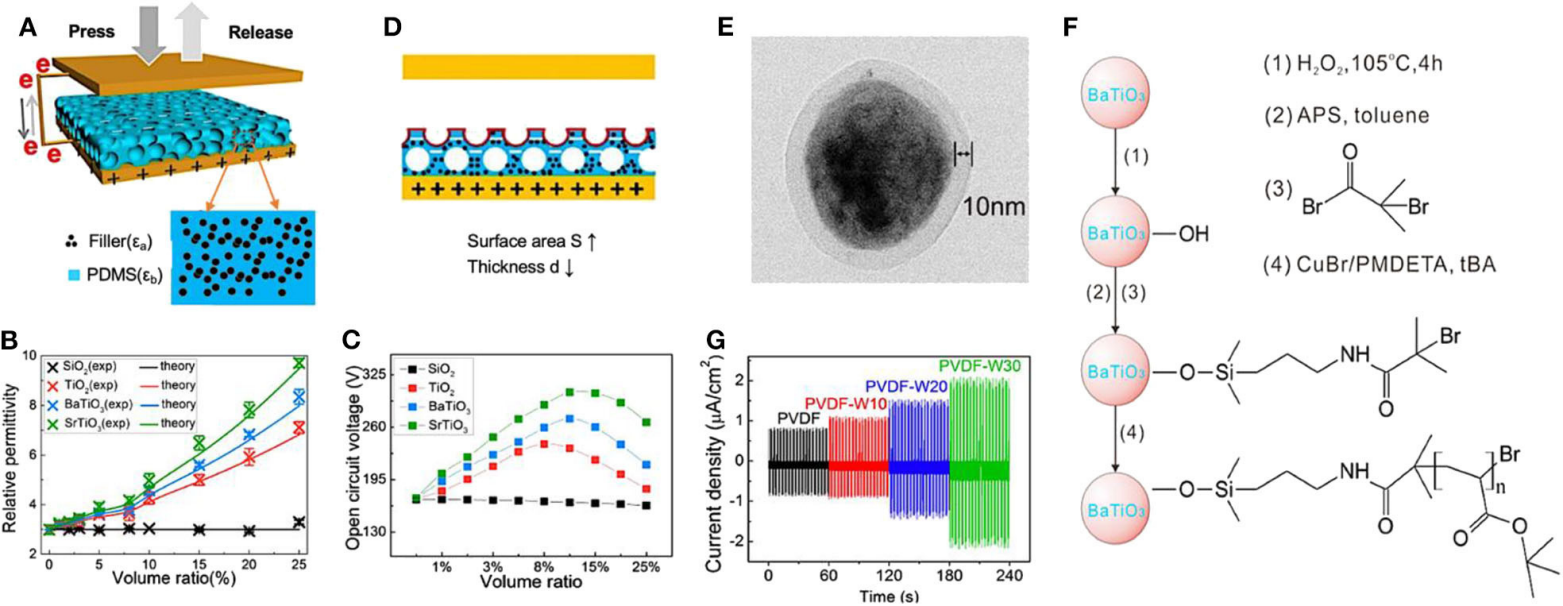
Schematic diagrams and output performance of nanoparticles-doped TENGs. **(A)** Structure and working principle of as-fabricated TENG. **(B)** The relative permittivity and **(C)** open circuit voltage of TENGs fabricated with different dielectric-nanoparticles-doped PDMS films. **(D)** Schematic diagram of TENG with sponge structure PDMS composite film. Reprinted with permission from Chen et al. (2016a). Copyright 2016 American Chemical Society. **(E)** TEM images of the core-shell-structured BaTiO_3_-PtBA nanoparticles. **(F)** Schematic diagram illustrating the preparation process of the core-shell-structured BaTiO_3_-PtBA nanoparticles. **(G)** Output current densities generated by the PVDF-based TENGs with different percentage of the BaTiO_3_-PtBA nanoparticles (ranging from 0 to 30%). Reprinted with permission from Du et al. (2018). Copyright 2018 American Chemical Society.

Nanoparticles with high permittivity are also doped inside the triboelectric materials to increase its dielectric constant, and therefore, to improve the output performance of the TENGs (Liu et al., 2017). Chen et al. filled the triboelectric materials, PDMS matrices, with high permittivity nanoparticles, such as SiO_2_, TiO_2_, BaTiO_3_, and SrTiO_3_ (Chen et al., 2016a), as shown in Figure 8A. The relative permittivity of these nanoparticles is demonstrated in Figure 8B. Among them, the TENGs doped with SrTiO_3_ particles generated the highest output voltage due to its highest relative permittivity of 300. Figure 8C displays the open-circuit voltage of TENGs fabricated with different dielectric-nanoparticles-doped PDMS films. The voltage was greatly enhanced after doping and reached a peak value of 305 V generated from the composite PDMS film containing 10 vol % SrTiO_3_ nanoparticles, while only 172 V can be obtained from the original PDMS. As a result, the peak voltage and current density for the TENG with composite PDMS film are 1.8 and 2.4 times as much as that of the original one.

In order to modulate the permittivity, a porous structure was formed inside the PDMS film by the process of simply and feasibly mixing and removing NaCl particles, as displayed in Figure 8D. When the TENG is pressed by a cyclic external force during the contact-and-separate movement, the triboelectric materials will shrink to minimum thickness, which increases the electrical output of the TENG due to the enlarged capacitance. Thus, the reason for introducing pores into triboelectric materials is that the effective thickness of the triboelectric materials can be reduced and the surface area can be enlarged simultaneously by forming sponge structure. However, adding pores into triboelectric materials, which can be regarded as filling particles with the permittivity of the air (even lower than the original triboelectric materials, PDMS), will have an adverse influence on the effective permittivity. The effective permittivity of PDMS film with sponge structure drops from 3 to 1.83 when the volume fraction of pores increases from 0 to 45%, verifying that filling with lower dielectric particles into the PDMS film causes a reduction in its effective permittivity. After comparing and balancing the three factors: effective thickness, top surface area, and permittivity of the PDMS film with sponge structure, the investigations found that the TENG based on the sponge PDMS friction film doped with 10 vol % SrTiO_3_ NPs and 15 vol % pores can provide an optimal output performance, reaching up to 9.06 μA/cm^2^ and 338 V in short-current density and open-circuit voltage, respectively. The as-modified TENG achieved over 5-fold power enhancement when compared with the TENG based on the pure PDMS film.

However, some researchers reported that the agglomeration effect, due to the high surface energy of nanoparticles doped into the triboelectric materials, might yield poor mechanical properties and high dielectric loss (Kim et al., 2007). Surface treatments should be applied in advance to reduce the surface energy and enhance the dispersity of nanoparticles to overcome the shortcoming of directly doping the high dielectric nanoparticle in the polymer matrix. Du et al. modified the surface of BaTiO_3_ nanoparticles with polymer polypoly(tert-butyl acrylate) (PtBA) and created core-shell-structured BaTiO_3_-PtBA nanoparticles by the atom transfer radical polymerization (ATRP) technique (Du et al., 2018). The transmission electron microscopy (TEM) images of the core-shell-structure BaTiO_3_ nanoparticles and the flow chart of the modification process are shown in Figures 8E,F. This PtBA shell can effectively reduce the surface energy of the nanoparticles in order to avoid aggregation in friction polymers, and therefore make the BaTiO_3_ nanoparticles maintain high flexibility with low dielectric loss. As demonstrated in Figure 8G, the TENG with BaTiO_3_-PtBA doped PVDF film generated a short-circuit current in the range from 1.1 to 2.1 μA/cm^2^, according to the weight percent of the BaTiO_3_-PtBA nanoparticles ranging from 0 to 30%, while the TENG with pure PVDF film generated only 0.8 μA/cm^2^. The as-modified nanocomposites have a high breakdown field, a high dielectric constant, and good mechanical properties. These favorable characteristics further improve the output performance of TENGs that are modified with the high dielectric nanoparticles doping method.

**Figure 9:**
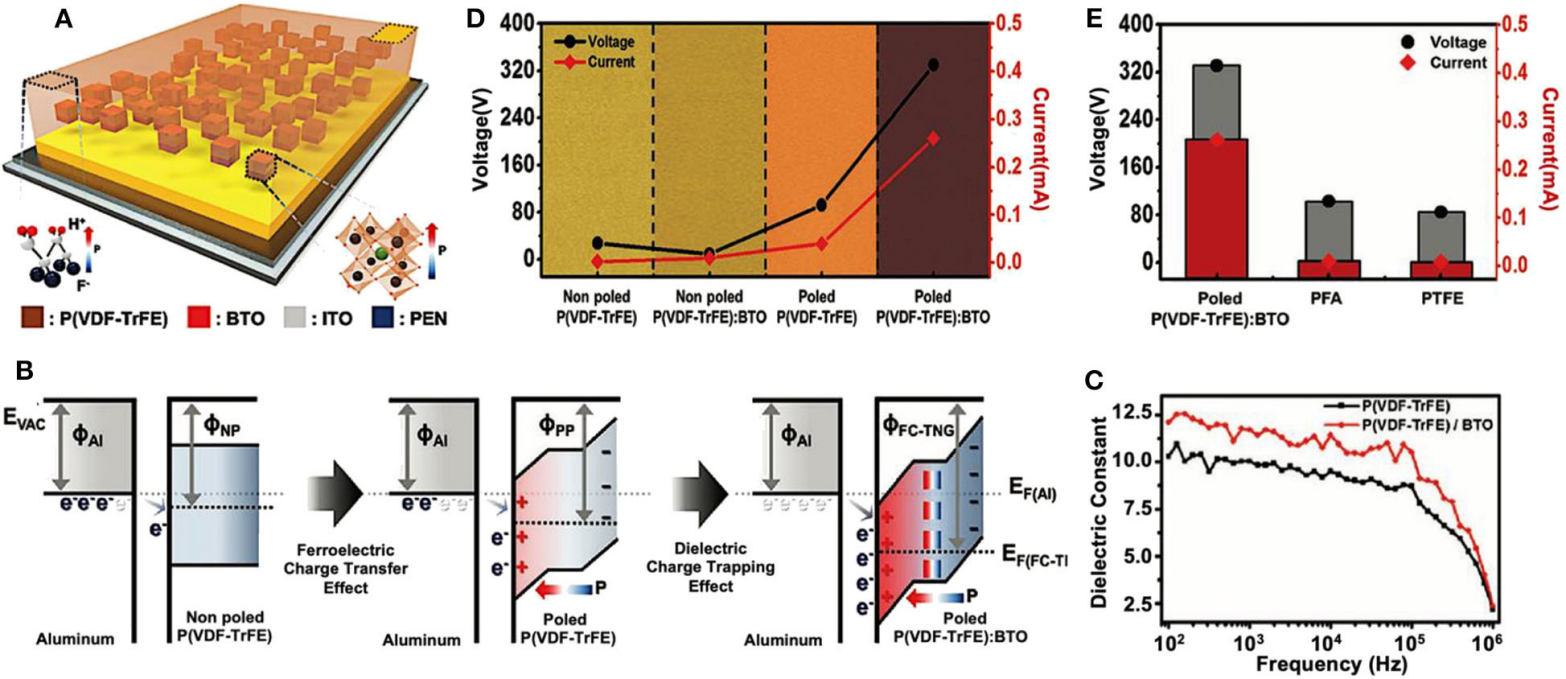
Schematic diagrams and output performance of a ferroelectric composite-based TENG with dielectric nanoparticles BTO. **(A)** Schematic representations of the structure of as-modified TENG. **(B)** The charge transfer behavior between two different material surfaces and its corresponding energy band modification. **(C)** The dielectric constant of P(VDF-TrFE) with and without dielectric nanoparticles BTO. **(D)** The output voltage and current of TENGs with different modification depending on with or without two effects: ferroelectric and dielectric. **(E)** Output performance comparison between ferroelectric composite-based TENG with BTO nanoparticles and TENGs with traditional polymer films. Reprinted with permission from Seung et al. (2017). Copyright 2017 John Wiley & Sons.

Aside from forming a sponge structure, there is another method, working together with nanoparticles doping, to further enhance the output performance of TENGs. Seung et al. applied both the relative permittivity effects and the polarization effects to modify the triboelectric materials (Seung et al., 2017). As displayed in Figure 9A, the triboelectric materials in this work are composed of poled ferroelectric copolymer matrix, poly(vinylidenefluoride-co-trifluoroethylene) [P(VDF-TrFE)] (Lee et al., 2015a,b), doped with high dielectric nanoparticles, BaTiO_3_ (BTO) (Wu et al., 2009; Kim et al., 2014). The poled ferroelectric P(VDF-TrFE) matrix could attract a large number of electrons from the opposite triboelectric material during the cyclic contact-and-separate movement. Moreover, the embedded BaTiO_3_ nanoparticles, with a high dielectric property, acting as a strong charge-trapping site of TENGs, can greatly increase the capacitance of triboelectric material (Wolters and van der Schoot, 1985). In this way, by embedding dielectric BaTiO_3_ nanoparticles inside the poled ferroelectric P(VDF-TrFE) copolymer matrix, TENGs with as-modified composite triboelectric materials can exhibit a dramatic increase in output performance (Zhong et al., 2015; Li W. et al., 2016; Wang et al., 2016). The charge transfer behavior between aluminum film and three different P(VDF-TrFE)-based surfaces, including non-poled P(VDF-TrFE), poled P(VDF-TrFE), and poled P(VDF-TrFE):BTO, are shown in Figure 9B. Among the three pairs, no significant charge transfer occurred between Al and non-poled P(VDF-TrFE). While in the pair of Al and poled P(VDF-TrFE):BTO, the band shifted steeply and a huge charge transfer occurred on the surface, owing to both the polarization of P(VDF-TrFE) (Shin et al., 2014) and the dielectric properties of the embedded nanoparticles. Figure 9C compared the dielectric constant between poled P(VDF-TrFE) and poled P(VDF-TrFE):BTO in order to visually display the electrical impacts brought from the introduction of high dielectric nanoparticles. For the whole measured range, the dielectric constant of the poled P(VDF-TrFE):BTO film was larger than the poled P(VDF-TrFE) film. The output voltage and current of TENGs based on different triboelectric materials, depending on with or without ferroelectric and dielectric effects, are shown in Figure 9D. Note that after embedding the BaTiO_3_ nanoparticles into the poled P(VDF-TrFE) rather than the non-poled P(VDF-TrFE), both the output voltage and current increased significantly, reaching nearly 360 V and 0.3 mA, respectively. That means the as-fabricated TENGs can only provide the maximum electrical output by introducing high dielectric nanoparticles and poling the matrix simultaneously. When compared with typical triboelectric material-based TENGs, as shown in Figure 9E, the as-fabricated TENG with poled P(VDF-TrFE):BTO film had a boosted output performance which was improved by about ~150 times.

Generally speaking, dielectric property modification is an effective and simple approach to fabricate high-performance TENGs. However, the percentage of nanoparticles embedded into the triboelectric material should be precisely controlled, since the introduction of new contents may affect other chemical or physical properties of the material itself including: surface effective area, material effective thickness and so on. In fact, the correlation between tribo-materials' dielectric properties and the output performance of TENGs is not clearly understood yet. Therefore, more investigations should be done to further improve the performance of energy harvesting.

## Functional Sublayers Insertion

Functional sublayers insertion is another effective way to increase the charge density of triboelectric materials and thus improve the output performance of the TENGs. By adding sublayers with different electrical properties into the TENG structure, the triboelectric charges in the bulk of triboelectric materials will redistribute to avoid the unnecessary loss of electrons. Furthermore, they will enlarge the friction layers' capacity of triboelectric electrons. Take the triboelectric electrons in a negative triboelectric material e.g., the original transport and storage process is as follows: when the electrons are accumulated on the contact surface after the cyclic contact between the positive and negative friction layers, the positive charges can be induced in the electrode. Due to this separate accumulation of opposite charges, a vertical upward electric field will be established between the friction surface and the bottom electrode. Therefore, the electrons will be transported deeper into the bulk of the triboelectric material, rather than stay on the friction surface through a drift process caused by the vertical electric field and a diffusion process caused by the concentration gradient of electrons. However, the loss of electrons may occur during the transport process. The positively charged ions or particles in the air may also be absorbed onto the friction surface, causing a decrease in the number of surface triboelectric electrons. Moreover, for the triboelectric electrons that transport deep into the bulk area, they may reach the bottom electrode and recombine with the induced positive charges. Furthermore, the triboelectric charges gathered on the surface will hinder the subsequent triboelectric charges from entering into the friction layer and cause a decline in charge density. In this case, one intuitive solution to the problem is to transport and trap triboelectric electrons into a specific middle layer of the triboelectric material, which is far from the friction surface. To store more charges, the sublayers used to trap the triboelectric electrons should have a higher dielectric constant. For the reasons above, adding multiple functional sublayers, including the charge transport layer with high electric conductivity and the charge storage layer with high dielectric property, etc., seems like a promising way to realize the electronic redistribution inside the triboelectric materials and therefore increase the charge density, which plays a vital role in improving the output performance of TENGs.

### Electrons Capture Layer Insertion

**Figure 10:**
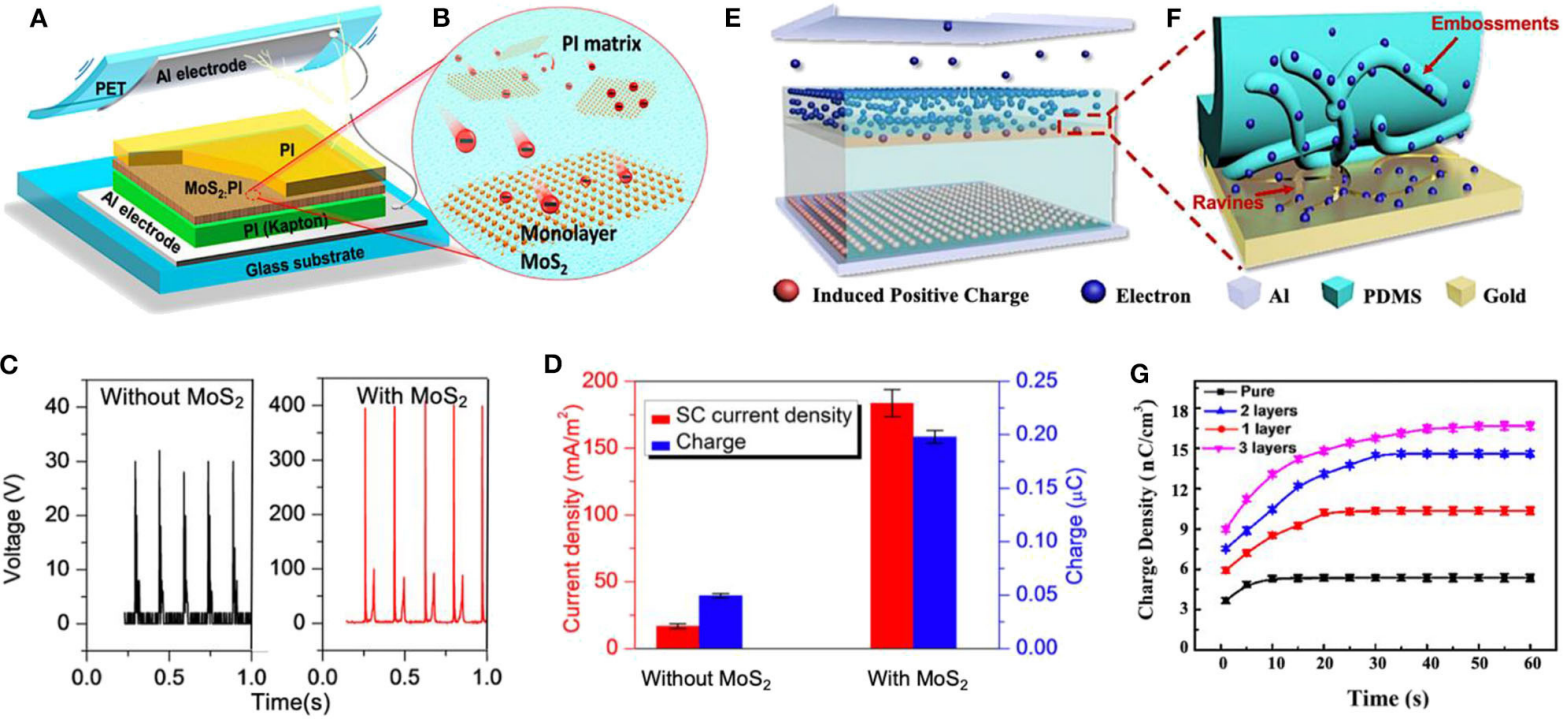
Schematic diagrams and output performance of TENGs with dielectric monolayers. **(A)** Schematic diagram of a vertical contact-separation mode TENGs based on the MoS_2_-inserted PI layer. **(B)** Schematic views of the electron transfer from the PI layer to the MoS_2_ monolayer. **(C)** Rectified open-circuit voltage of the TENGs without and with MoS_2_ monolayer. **(D)** Short-circuit current density and the amount of charge generated during a press-release cycle for the TENGs with and without monolayer. Reprinted with permission from Wu et al. (2017). Copyright 2017 American Chemical Society. **(E)** Illustrations of electrons' drift process in the TENG based on Au film. **(F)** Schematic diagram of electrons tunnel from PDMS to the Au film. **(G)** Transfer charge densities of TENGs. Reprinted with permission from Lai et al. (2018). Copyright 2018 American Chemical Society.

Typically, monolayer molybdenum disulfide (MoS_2_), similar to the reduced graphene oxide (GO) sheet, is a competitive candidate as the charge-trapping agent due to its quantum confinement effect, large specific surface area, and appropriate energy level (Liu et al., 2012; Shin et al., 2016). Therefore, Wu et al. introduced 2D MoS_2_ monolayer sheets inside the triboelectric materials of TENG in an attempt to dramatically enhance its output performance, as displayed in Figure 10A (Wu et al., 2017). In the structure of TENG, a PI layer and Al film were chosen as the negative and the positive triboelectric material. Based on this, a MoS_2_ monolayer sheet was inserted into the bulk of the PI material by spin-coating and subsequent imidization (Wu et al., 2011), to create a uniform MoS_2_-inserted PI layer. During the cyclic contact-and-separate movement of as-fabricated TENG, the triboelectric electrons generated on the surface of the PI may be transferred into the MoS_2_ monolayer. Furthermore, they may also be stored inside the negative friction layer rather than on its surface; the aforementioned transfer and storage of electrons within this system are displayed in Figure 10B. This phenomenon can be attributed to the high charge-trapping property of the MoS_2_ monolayer. Therefore, the static electron density on the negative triboelectric surfaces is decreased, increasing the gap between the positive and the negative charges. The high electron-capture properties of the monolayer MoS_2_ can weaken the air breakdown effect, the recombination process between triboelectric electrons, and the later absorbed positive charges, which can both cause the loss of generated triboelectric electrons. Additionally, the monolayer MoS_2_ can also suppress the drift and the diffusion effects of electrons. The electrons in the friction layer serve as an electrostatic induction source for the electricity generation process of the TENGs, which has a great impact on the output performances of TENGs. As a result, the electrical output performance of as-fabricated TENG can be enhanced by the dramatic increase in the density of triboelectric electrons.

To verify the impact of MoS_2_ monolayer insertion, a vertical contact-separation mode TENG containing MoS_2_ monolayer as an electron-acceptor layer was fabricated. Figure 10C shows that the open-circuit voltage of the TENG without MoS_2_ monolayer is about 30 V, however, that of the TENG equipped with MoS_2_ is as high as 400 V. Furthermore, the as-fabricated TENG exhibited a dramatic enhancement in its short-circuit current density and the amount of charge generated by the TENG, as displayed in Figure 10D. The total amount of charge generated by TENG with MoS_2_ monolayer reached ~0.2 μC, which was much larger than that generated by using a TENG without MoS_2_ monolayer with a value of 20.05 μC. Moreover, the peak power density of modified TENG was as large as 25.7 W/m^2^, which is 120 times larger than that of the pristine TENG. These performance enhancements are attributed to the highly efficient electrons-captured ability of some novel 2D materials, which can provide direction to a frontier inactive material selection and device structure design for high efficiency triboelectric devices.

### Electrons Trapping Layer Insertion

In addition to a MoS2 monolayer, the conductive materials serving as sublayers have also been reported. These materials are normally inserted in a bulk triboelectric material as transport layers and form sandwich structures. Lai et al. embedded ravines, gullies, and crisscrossed Au layers into the near-surface region of the negative triboelectric material of PDMS, as shown in Figure 10E (Lai et al., 2018). In order to obtain the PDMS triboelectric material equipped with the Au layer, gold was coated on the surface of PDMS via magnetron sputtering deposition. It was determined, through theoretical analysis, that when the negative triboelectric material, PDMS, captures the triboelectric charges from the positive triboelectric material, Al film, the charges drift from the surface to the internal bulk of the triboelectric material. This can be credited to the diffusion process caused by the concentration gradient of electrodes and the strong electric field between the friction surface and the bottom electrode. The Au layers applied in the work act as the passageways and trapping agent of the triboelectric charges during the drifting process. When the triboelectric charges reach the interface between the PDMS and the Au layer, owing to the strong electric field, the triboelectric charges will tunnel from the PDMS to the gold. In this way, the internal-space-charge zone is built. Moreover, the existence of ravines and gullies in PDMS and crisscrosses in the Au layers jointly benefit from the tunneling of charges. Owing to the existence of ravines in the gold layer, formed by plasma treatment, there are many embossments similar to those in mountain chain formation via PDMS, as shown in Figure 10F. As we know, for a charged object, the charge surface density is proportional to the curvature. Thus, electrons will assemble in the embossments, resulting in an increase of the local electric field strength. Accordingly, the increased strength of the local electric field leads to an increase in the probability of tunneling. Through embedding ravines and gullies, the crisscrossed gold layer has an improved charge storage capability and enhanced storage depth of the triboelectric charge, which leads to the high output performance of TENG. Figure 10G displays the transfer charge densities of TENGs, where the charge density of the TENG with Au trapping layer increases to 16.8 nC/cm^2^ which is ~3 times higher than that of the pure TENG without the Au layer. TENGs equipped with multiple gold layers were built in the work to investigate the changing tendency of triboelectric charge densities. With the increasing number of inserted gold layers, from 0 to 4, the value of transfer charge density is about 5.4, 9.7, 14.5, 16.7, and 16.8 nC/cm^2^, respectively, showing a gradual steady tendency at around 16.8 nC/cm^2^ when the number of layers reaches three. This observation implies that the charges cannot drift much, because they are being restricted by the depth of the drift and depressed by the product of the probabilities of tunneling in each interface. Overall, this study paves a novel way to enhance the charge density of the triboelectric materials and, therefore, improve TENG's output performance.

### Multiple Layers Structure

**Figure 11:**
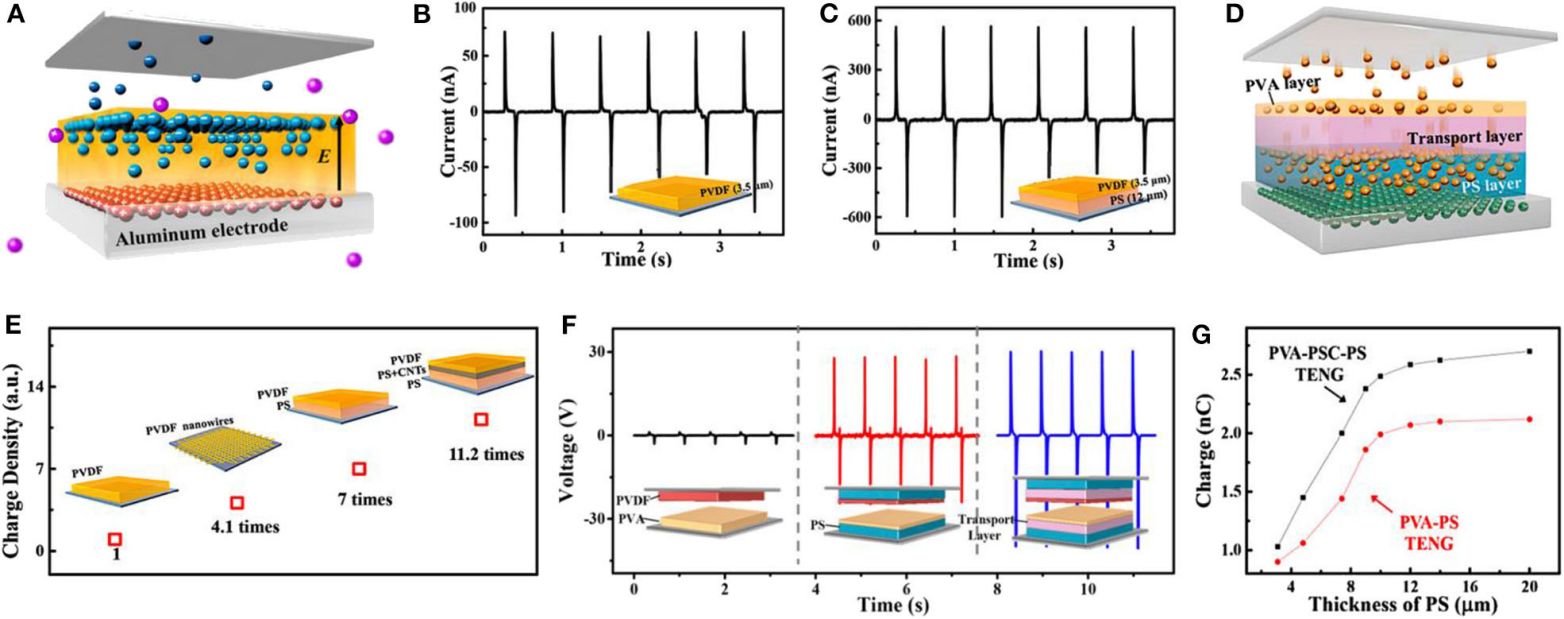
Schematic diagrams and output performance of TENGs with multiple functional sublayers. **(A)** Schematic diagram of the transport process of triboelectric electrons in the negative triboelectric material of TENG. **(B,C)** The output comparation of short current generated from TENG **(B)** without and **(C)** with PS dielectric sublayer. **(D)** Improvement effects of different composite friction layer structure. Reprinted with permission from Cui et al. (2016). Copyright 2016 American Chemical Society. **(E)** Schematic of the electrons transport process of TENG with three-sublayers structure. **(F)** The voltage of TENG with different sublayers applied in both the positive and negative triboelectric materials. **(G)** The charge capacity of TENGs fabricated with different thicknesses of PS dielectric layers. Reprinted with permission from Cui et al. (2018). Copyright 2018 American Chemical Society.

Based on the electrons trapping layer insertion method mentioned above, a composite multiple-layer structure was built in the triboelectric materials, in order to adjust the internal distribution of the triboelectric charges and therefore improve the output performance of TENGs. The structure was formed by the stacking of a charge capture layer, a charge transport layer, and a charge storage layer from the friction surface to the bottom electrode. Among these three sublayers, the charge capture layer was used as the fundamental friction layer that was equipped in all of the structures of TENGs demonstrated in Figure 11A. Furthermore, the charge capture layer was used for robbing charges effectively from the opposite friction layer during the cyclic contact-separate-movement. In contrast, the charge transport sublayer was inserted to transport the generated triboelectric charges on the surface to deeper inside the material. Finally, the charge storage layer was added in order to hold more charges in the triboelectric materials. For these purposes, the transport sublayer should use materials with high conductivity in order to allow for triboelectric charges to pass through this layer quickly and hardly reside in it. This passageway for charge can not only help the generated triboelectric charges on the surface transport deeper into the bulk area, but also weaken the repulsive force for the later coming charges as well. As for the charge storage sublayer, it should be made of materials with lower carrier mobility, low intrinsic carrier concentration, and rich defects, which can all contribute to a larger charge capacity. As we all know, increasing the triboelectric charge density of a friction layer is one of the most basic approaches to improve the electrical output performance of TENGs.

On the basis of the theoretical analysis, Cui et al. fabricated TENGs based on the above three-sublayers structure (Cui et al., 2016). The negative and positive friction layers of TENGs fabricated in this work were polyvinylidene fluoride (PVDF) and aluminum foils. Firstly, the PS dielectric sublayer was added into the negative triboelectric material, as a charge storage layer between the bottom aluminum electrode and PVDF friction layer. The intrinsic carrier density and electron mobility of PS are both smaller than that of PVDF, due to its abundant trap levels of electrons, making PS one of the most suitable dielectric materials for acting as a storage layer. The short-circuit currents performances, compared in Figures 11B,C, indicate that the TENG with the PS sublayer demonstrated a dramatically enhanced short-circuit current value with nearly 600 nA, which is over 8 times greater than that of the TENG without the sublayer. The triboelectric charge density of the triboelectric material with this new structure is 7 times larger than that with the pure PVDF layer, as shown in Figure 11D. To further enhance the output performance of TENGs, a charge transport layer made of a small number of carbon nanotubes was inserted between the PVDF friction layer and the PS storage layer, in order to increase the conductivity of the near-surface area in triboelectric material. The transport sublayer is formed by adding a small number of carbon nanotubes (CNTs) into a PS or poly(vinyl alcohol) (PVA) layer. In this way, the transport process of the triboelectric charge in the negative triboelectric materials was improved as follows: triboelectric charges from the positive friction layer was first captured by the outermost PEDF layer and entered the friction surface. Under the force of a built-in electric field between the negative triboelectric charges on the friction surface and the positive induced charges in the electrode, these charges migrated deeper into the bulk area through the CNTs-based PS layer and finally remained in the PS dielectric layer. This further introduction of the charge transport layer can increase the triboelectric charge yield effectively. And in this way, the total charge quantity in the friction layer could be increased again by a factor of 1.6. Overall, these multiple-layer structures of negative triboelectric material provided a factor of 11.2 improvement of the charge density produced from as-fabricated TENG when compared to the original non-modified one.

Based on the previous work, it can be speculated that the introduction of the charge transport and storage sublayers, which has been proved to be effective in improving the electron storage capacity for the negative triboelectric materials, may also take effect in the positive triboelectric materials. As demonstrated in Figure 11E, Cui et al. also introduced a similar multilayer structure to the positive triboelectric material, PVA layer (Cui et al., 2018). The three-sublayers structure are, in this case, from the friction surface to the bottom electrode, a 2 μm thick PVA layer, a 5 μm thick PS-doped 0.7 wt % CNTs layer (PSC), and PS layer ranging from 3 to 20 μm, working as the positive charge capture layer, the charge transport layer and the storage layer, respectively. Actually, to achieve the optical output performance of TENG, the structure with three collaborative sublayers may well be applied to both the negative and positive friction layer. Therefore, three TENGs equipped with different sublayers are built to investigate their output performance, including a TENG with a PVA positive friction layer and a PVDF negative friction layer, the second TENG with the same pair of friction layer and an inserted PS storage layer, the third TENG added both the PSC transport layer and PS storage layer into the same friction pair. The schematic diagram of these structures and the output voltage are shown in Figure 11F. The TENG with multiple functional sublayers exhibited a dramatic enhancement on output performance when compared to the original TENG. Moreover, by introducing the PSC layer into the positive friction layer, the charge capacity of TENG could be improved by 3.7-fold, from 0.37 to 2.7 nC/cm^2^, as displayed in Figure 11G. By properly applying this three-sublayers structure to both positive and negative friction layers, the charge capacity of the designed TENG would be able to improve by 16.5-fold, which results in an outstanding promotion in the performance of the as-fabricated TENG.

Furthermore, in this multiple-layer structure, the thickness of each inserted sublayer has a vital relationship with the total charge capacity of the triboelectric materials and their inner charge distribution. Figure 11G shows the charge capacity of TENG with the bottom PS charge storage layer of different thicknesses, ranging from 3 to 20 μm, while the devices' top PVA layers are all 2 μm and PSC layers are 5 μm. From the output performance test, it can be found that with the increase of the PS layer's thickness, the charge capacity reveals an initial trend of rapid rising and finally becoming stable finally, at about 12 μm. The same trend of output performance can also be found in the structure with a charge capture layer or transport layer of changing thickness. The charge capacity of the inserted capture layer PVA and the inserted transport layer PSC continues to increase with the increase of the thickness of each layer, respectively, but the increasing rate decreases gradually. This means that an excess of thickness for these sublayers will not supply more extra effective storage space for the triboelectric charges. When comparing the performance difference between devices with (shown in black line) and without (shown in red line) a charge transport layer in this multiple-layer structure, it can also be found that the thicker the PS layer, the greater the influence of the charge transport layer. This is because, for a PVA–PSC–PS three-layers structure TENG with a thicker PS layer, a greater proportion of positive charges is separated from the surface by the transport layer. Therefore, the function of the inserted charge transport layer certainly becomes more obvious.

In general, adding additional sublayers with different electrical properties into the TENG structure yields an active intervention to the electrons transport and storage process; therefore, functional sublayers insertion is a promising way to improve the output performance of the TENGs. The triboelectric charges in the bulk of triboelectric materials were redistributed, in order to avoid the unnecessary loss of triboelectric electrons. Furthermore, this enlarges the friction layers' capacity of triboelectric electrons. However, the mechanism of the collaborative effect from the inserted sublayers, inner interfaces, and the triboelectric material, which, respectively, enhances the effective capture, transfer and storage of triboelectric charges has not yet been well-studied. Accordingly, future studies in this field will have great guiding significance for improving the performance and stability of TENGs.

## Conclusions

In this review, surface chemistry was systematically introduced to promote the mechanical to electrical energy conversion via triboelectric nanogenerators. Through chemical modifications and approaches, triboelectric materials' surface charge densities have been proven to be modulated and thus the output performances of TENGs are improved. Table 1 systematically summarizes the performance enhancements and mechanical durability of each chemical-modified TENGs. Methods that have been discussed in this review include functional groups grafting, ion implantation and decoration, dielectric properties engineering, and sublayers insertion. TENGs with broadened material choices, diversified operation modes and structural design have fully displayed the advantages in a variety of application scenarios, comparing with other mechanical energy harvesting techniques. Many challenges are waiting to be overcome to advance the field of surface chemistry for high-performance TENGs, as follows.

1. Enhance the chemical stability of the surface chemical functional group grafting. Functional group grafting is a straightforward, cost-efficient, and easy-to-implement way to efficiently enhance the output performance of TENGs. Although that may be true, the results may lose its effectiveness if the surface is polished or worn out during the friction since the modification just takes place on the surfaces rather than deep into the bulk of triboelectric materials. To overcome this difficulty and limitation, further improvements should be found to enhance stability and effective duration of the method.
2. Reduce the cost of ion injection and decoration methods for further scaling up. The ion injection and decoration methods provide effective approaches for improving the TENG's output performance with relatively high stability. However, the fabrication processes are relatively complicated and the instruments used during the processes, such as air-ionization guns, add extra cost. It may bring obstacles for further scaling up. Consequently, more flexible and cost-efficient techniques should be explored for ion implantation promotion.
3. Deeply explore the mechanisms of dielectric engineering. Although the dielectric property modification is experimentally proven to be an effective and simple approach for obtaining high-performance TENGs, the correlation between dielectric properties of triboelectric materials and output performance of as-fabricated TENGs is not clearly unveiled yet. Further investigation should be done to explore the mechanisms of dielectric engineering and reveal the coefficient between embedded nanoparticles and other chemical and physical properties of the material itself, therefore optimizing the dose of dopants for high-performance TENGs.
4. Investigate the inner mechanism of functional sublayers insertion. Functional sublayers insertion is a promising way to improve the output performance of the TENGs because it can provide an active intervention to the electrons transport and storage process. Nevertheless, the mechanism of the collaborative effect from the inserted sublayers, inner interfaces, and the triboelectric material has not yet been well-studied. Thus, further studies investigating the inner mechanism will have great guiding significance to further improve the output performance of TENGs.
5. Incorporation of chemical and physical modifications. The synergy of chemical and physical modifications could be an effective approach to build up a high-performance TENGs. Applying them to a material of interest can not only increase the effective contact area between the two triboelectric surfaces by various surface morphologies, but also increase the gap between the two triboelectric materials' ability to gain or lose electrons as well. Therefore, more attempts should be done to apply both chemical and physical modifications together to triboelectric materials or to explore new techniques that can modify surface morphologies and chemical properties simultaneously.
6. Seek for possible biochemical approaches. Chemical and biological approaches are usually highly correlated with each other. By introducing biomaterials as novel triboelectric materials, like silk fibroin film, TENG may have some satisfying superiorities, such as flexible, stretchable, bio-friendly and preferably transparent. Therefore, it should be wearable (or even implantable) to the human body. Through applying proper chemical modifications to these biomaterials, higher electrical performance and working stability can also be achieved, making TENG an efficient and reliable power source with wider application prospects.
7. Explorations of versatile TENG-based self-powered devices. Both theoretical calculations and experimental endeavors are highly in need for promoting existing materials or potentially discovering new materials. which are not only with high triboelectric properties but also compatibility with various application scenarios, which seem to be a basis for the development of versatile TENG-based self-powered devices.

**Table 1:** A summary of typical chemical modification methods for TENGs.

**Tribo-materials**	**Modification method**	**Output performance enhancement*****^b^***				**Duration*^c^***	**References**
		**Charge**	**Voltage**	**Current**	**Power**		
Cl-PET, PEI(b)-PET	Self-assembled monolayer	-	19.80 *^a^*	21 *^a^*	-	5,000	Shin et al., 2017
PDMS, ITO	UVO-irradiation	15.88	11.97	17.46	-	20,000	Yun et al., 2015
PMMA^−^ fiber, Cu	Electrospinning	-	1.28 *^a^*	147 *^a^*	585 *^a^*	125,000	Busolo et al., 2018
FEP, Al	Ion Injection	5	5	3.33	25	400,000	Wang et al., 2014
FEP, Kapton	Ion Irradiation	2.32 *^a^*	-	-	35,999	-	Li et al., 2020
FOTS-PDMS, Li^+^ film	Ion Absorption	-	2.40	5.25	20	50,000	Park et al., 2017
AlO_X_-PDMS, PDMS	Sequential Infiltration Synthesis	8	6.67	6.33	-	-	Yu et al., 2015
BaTiO_3_-doped(PVDF-TrFE), Al	High Dielectric Doping	2.52	2 *^a^*	5 *^a^*	150	-	Seung et al., 2017
PI/MoS_2_:PI/PI, Al	Caption-Layer Insertion	3	12.33	6 *^a^*	120	-	Wu et al., 2017
PDMS-Au layer, Al	Trapping-Layer Insertion	4	-	-	-	-	Lai et al., 2018
PVA-PSC-PS, PVDF-PSC-PS	Multiple-Layer Structure	16.5	9 *^a^*	9 *^a^*	-	-	Cui et al., 2018

a * Estimated value*.

b * Multiple of Enhancement*.

c * Testing Cycle of Duration*.

Overall, the utilization of surface chemistry opens an emerging and effective route to build up high-performance triboelectric nanogenerator as a pervasive energy solution in the upcoming era of the Internet of Things. Challenges coexist with opportunities, and much more research efforts remain desired to improve surface chemical modification with the goals of improved stability, robustness, scalable and advanced surface modification. We anticipate that the wide application of surface chemistry can contribute largely to develop high-performance TENGs as both sustainable power sources and active sensors.

## Author Contributions

JC: supervision, visualization, and funding acquisition. JC and JX: conceptualization and writing-original draft. JX, JC, YZ, and AN: writing-review and editing. All authors contributed to the article and approved the submitted version.

## Conflict of Interest

The authors declare that the research was conducted in the absence of any commercial or financial relationships that could be construed as a potential conflict of interest.
